# Stable spike clusters for the precursor Gierer–Meinhardt system in $\mathbb {R}^2$

**DOI:** 10.1007/s00526-017-1233-6

**Published:** 2017-09-22

**Authors:** Juncheng Wei, Matthias Winter, Wen Yang

**Affiliations:** 1 Department of Mathematics, University of British Columbia, Vancouver, BC V6T 1Z2 Canada; 2 Department of Mathematics, Brunel University London, Uxbridge, UB8 3PH UK; 3 Department of Applied Mathematics, Hong Kong Polytechnic University, Hung Hom, Kowloon, Hong Kong

**Keywords:** 35B35, 92C15, 35B40, 35B25

## Abstract

We consider the Gierer–Meinhardt system with small inhibitor diffusivity, very small activator diffusivity and a precursor inhomogeneity. For any given positive integer *k* we construct a spike cluster consisting of *k* spikes which all approach the same nondegenerate local minimum point of the precursor inhomogeneity. We show that this spike cluster can be linearly stable. In particular, we show the existence of spike clusters for spikes located at the vertices of a polygon with or without centre. Further, the cluster without centre is stable for up to three spikes, whereas the cluster with centre is stable for up to six spikes. The main idea underpinning these stable spike clusters is the following: due to the small inhibitor diffusivity the interaction between spikes is repulsive, and the spikes are attracted towards the local minimum point of the precursor inhomogeneity. Combining these two effects can lead to an equilibrium of spike positions within the cluster such that the cluster is linearly stable.

## Introduction

In 1952, Turing [16] studied how pattern formation could start from a state without patterns. He explained the onset of pattern formation by a combination of two properties of the system:

(i) presence of spatially varying instabilities
(ii) absence of spatially homogeneous instabilities.

Since Turing’s pioneering work many models have been proposed and studied to explore the so-called *Turing diffusion-driven instability* in reaction–diffusion systems to understand biological pattern formation. One of the most popular of these models is the Gierer–Meinhardt system [5, 12, 13], which in two dimensions can be stated as follows:

1.1 $$\begin{aligned} {\left\{ \begin{array}{ll} A_t=\varepsilon ^2\Delta A-\mu A+\frac{A^2}{H}, \quad &{}\text{ in } \Omega , \\ \tau H_t=D\Delta H-H+A^2, \quad &{}\text{ in } \Omega ,\\ \frac{\partial A}{\partial \nu }=\frac{\partial H}{\partial \nu }{=0}, \quad &{}\text{ on } \partial \Omega . \end{array}\right. } \end{aligned}$$

We assume that the diffusivities $\varepsilon$ and *D* are small positive constants satisfying $0<\varepsilon ^2\ll D\ll \frac{1}{\log \frac{\sqrt{D}}{\varepsilon }}\ll 1$ and $\tau$ is a nonnegative constant which is independent of $\varepsilon$. Further, $\Omega \subset \mathbb {R}^2$ is a smooth bounded domain and $\frac{\partial }{\partial \nu }$ denotes the outward normal derivative at a point on its boundary $\partial \Omega$. In this paper we assume that $\Omega =B_R$ is a disk around the origin with radius *R*. For the standard Gierer–Meinhardt system it is assumed that $\mu (y)\equiv 1$. In this study we consider a precursor inhomogeneity $\mu (|y|)$ which is a positive, rotationally symmetric and sufficiently smooth function of the spatial variable *y* defined in the domain $\Omega$.

The main idea underpinning these stable spike clusters is the following: due to the small inhibitor diffusivity the interaction between spikes is repulsive and the spikes are attracted towards the local minimum point of the precursor inhomogeneity. Combining these two effects can lead to an equilibrium of spike positions within the cluster such that the cluster is linearly stable. The repulsive nature of spikes has been shown in [4]. The attracting feature of the local minimum of a precursor has been established in [17].

Problem (1.1) without precursor has been studied by numerous authors. For the one-dimensional case in a bounded interval $(-1,1)$ with Neumann boundary conditions, the existence of symmetric *N*-peaked solutions (i.e. spikes of the same amplitude in leading order) was first established by Takagi [15]. The existence of asymmetric *N*-spikes was first shown by Ward and Wei [18] and Doelman et al. [3] independently. For symmetric *N*-peaked solutions, Iron et al. [8] studied the stability by using matched asymptotic expansions while Ward and Wei [18] later studied the stability for asymmetric *N*-spikes. Existence and stability for symmetric spikes in one spatial dimension was then established rigorously by the first two authors [23].

For the Gierer–Meinhardt system in two dimensions, the first two authors rigourously proved the existence and stability of multiple peaked patterns for the Gierer–Meinhardt system in the weak coupling case and the strong coupling case for symmetric spikes [20–22]. Here we say that the system is in the weak coupling case if $D\rightarrow \infty$ as $\varepsilon \rightarrow 0$ and in the strong coupling case if the parameter *D* is a finite constant independent of $\varepsilon$. For more results and background on the Gierer–Meinhardt system, we refer to [26] and the references therein.

In fact, already in the original Gierer–Meinhardt system [5, 12, 13], the authors have introduced precursor inhomogeneities. These precursors were proposed to model the localisation of the head structure in the coelenterate *Hydra*. Gradients have also been used in the Brusselator model to restrict pattern formation to some fraction of the spatial domain [7]. In this example, the gradient carries the system in and out of the pattern-forming region of the parameter range (for example across the Turing bifurcation). Thus it restricts the domain where peak formation can occur. A similar localisation effect has been used to model segmentation patterns for the fruit fly Drosophila melanogaster in [6, 11].

In [24] the existence and stability of *N*-peaked steady states for the Gierer–Meinhardt system with precursor inhomogeneity has been explored. The spikes in these patterns can vary in amplitude and have irregular spacing. In particular, the results imply that a precursor inhomogeneity can induce instability. Single-spike solutions for the Gierer–Meinhardt system with precursor including spike dynamics have been studied in [17].

Recently, the first two authors in [27] studied the Gierer–Meinhardt system with precursor in one spatial dimension and proved the existence and stability of a cluster, which consists of *N* spikes approaching the same limiting point. More precisely, they consider the existence of a steady-state spike cluster consisting of *N* spikes near a nondegenerate local minimum point $y_0$ of the inhomogeneity $\mu (y)$, i.e., $\mu '(y_0)=0$, $\mu ''(y_0)>0$, where $y\in (-1,1),\, y_0\in (-1,1)$. Further, they show that this solution is linearly stable.

Now we consider this problem in two dimensions. We shall study the existence and stability of positive *k*-peaked steady-state spike clusters to (1.1). For simplicity, we shall study the steady-state problem for positive solutions of (1.1) in the disk $B_R$ around the origin with radius *R*, which can be stated as follows:

1.2 $$\begin{aligned} {\left\{ \begin{array}{ll} \varepsilon ^2\Delta A-\mu (|y|)A+\frac{A^2}{H}=0,\quad &{}\text{ in } B_R,\\ D\Delta H-H+A^2=0,\quad &{}\text{ in } B_R,~\\ \frac{\partial A}{\partial \nu }=\frac{\partial H}{\partial \nu }=0, \quad &{}\text{ on } \partial B_R~, \end{array}\right. } \end{aligned}$$

where $\frac{\partial }{\partial \nu }$ denotes the outward normal derivative at a point on $\partial B_R$.

Inspired by the work [1], where the authors constructed multi-bump ground-state solutions and the centres of these bumps are located at the vertices of a regular polygon, while each bump resembles, up to translation and multiplication with a constant amplitude, the unique radially symmetric solution of

1.3 $$\begin{aligned} \Delta w-w+w^2=0~\mathrm {in}~\mathbb {R}^2,\quad 0<w(x)\rightarrow 0~\mathrm {as}~|x|\rightarrow \infty , \end{aligned}$$

in this paper we shall prove the existence and stability of a spike cluster located near a nondegenerate minimum point of the precursor such that the positions of the spikes form a regular polygon. We note that the presence of such patterned steady state configurations appears driven by the smallness of the relative size $\sigma ^2=\varepsilon ^2/D$ of the diffusion rates of the activating and inhibiting substances. However, there is some difference between our problem and the one considered in [1]. Here, we also need to take into consideration the precursor $\mu (|y|)$ and further assume that the inhibitor diffusivity *D* is very small. After introducing the transformation

$$\begin{aligned} y=\varepsilon x,~{\hat{A}}(x)=\frac{1}{\xi }\frac{\varepsilon ^2}{D}A(\varepsilon x), ~{\hat{H}}(x)=\frac{1}{\xi }\frac{\varepsilon ^2}{D}H(\varepsilon x), \end{aligned}$$

and dropping hats, Eq. (1.2) becomes,

1.4 $$\begin{aligned} {\left\{ \begin{array}{ll} \Delta A-\mu (|\varepsilon x|)A+\frac{A^2}{H}=0,\quad &{}\text{ in }~B_{R/\varepsilon },\\ \Delta H-\sigma ^2H+\xi A^2=0,\quad &{}\text{ in }~B_{R/\varepsilon },\\ \frac{\partial A}{\partial \nu }=\frac{\partial H}{\partial \nu }=0, \quad &{}\text{ on }~\partial B_{R/\varepsilon }, \end{array}\right. } \end{aligned}$$

where the explicit definition of $\xi$ will be given in (3.6).

Our first result on the existence of *k*-spike clusters is the following:

### Theorem 1.1

(Existence of *k*-spike clusters). Let $k\ge 2$ be a positive integer. We assume $\mu =\mu (|y|)\in {C^3(B_{R})}$ be a positive, radially symmetric function and $\mu (0)=1,\mu '(0)=0,\mu ''(0)>0$, where $\mu '$ denotes the radial derivative. Then, for

$$\begin{aligned} \max \left( \frac{\varepsilon }{\sqrt{D}},D\log \frac{\sqrt{D}}{\varepsilon }\right) \rightarrow 0, \end{aligned}$$

problem (1.2) has a *k*-spike cluster solution which concentrates at 0. In particular, it satisfies

1.5 $$\begin{aligned} A_{\varepsilon }(y)\sim \sum _{i=1}^k\frac{\xi D}{\varepsilon ^2}w\left( \frac{y}{\varepsilon }-q_i\right) ,~ H_{\varepsilon }(q_i)\sim \frac{\xi D}{\varepsilon ^2}, \end{aligned}$$

where $\xi$ is given in (3.6) and $q_1,\ldots ,q_k$ are the vertices of a *k* regular polygon. Further, $\varepsilon q_i\rightarrow 0,~i=1,\ldots ,k$.

### Remark 1

Here the assumption on the value of $\mu (0)=1$ is introduced to make the computation and representation convenient. Without loss of generality we can always apply some scaling transformation for the solution $(A_{\varepsilon },H_{\varepsilon })$ to achieve the assumption $\mu (0)=1$.

### Remark 2

The limit

$$\begin{aligned} \max \left( \frac{\varepsilon }{\sqrt{D}},D\log \frac{\sqrt{D}}{\varepsilon }\right) \rightarrow 0 \end{aligned}$$

is equivalent to the two simultaneous limits

$$\begin{aligned} \frac{\varepsilon }{\sqrt{D}}\rightarrow 0 \end{aligned}$$

and

$$\begin{aligned} D\log \frac{\sqrt{D}}{\varepsilon }\rightarrow 0. \end{aligned}$$

The first limit

$$\begin{aligned} \frac{\varepsilon }{\sqrt{D}}\rightarrow 0 \end{aligned}$$

means that the diffusivity of the activator *A* is asymptotically of a higher order than the diffusivity of the inhibitor *H*. If this is not satisfied the pattern observed will no longer have a spike profile.

For the second limit

$$\begin{aligned} D\log \frac{\sqrt{D}}{\varepsilon }\rightarrow 0 \end{aligned}$$

to hold it is necessary that $D\rightarrow 0$. (In fact, this is the condition which occurs in the case of one spatial dimension.) Here, for two spatial dimensions there is a second factor $\log \frac{\sqrt{D}}{\varepsilon }$ which tends to infinity due to the first limit. The second factor appears due to the logarithmic singularity of the Green’s function, see (3.4). The second limit is exactly the condition which guarantees that the spikes form a cluster, i.e. their distances tend to zero, see (3.1), (3.2).

### Remark 3

The exact scaling of $q_i$ follows from the balancing condition (4.11) with the radius $R_\varepsilon$ defined in (3.2). In this balancing condition there are two contributions: the first comes from the interactions of neighbouring spikes, the second stems from the precursor inhomogeneity.

*Key steps in the proof of Theorem* 1.1:

### Proof

Step 1. In Sect. 3 we use an ansatz of an approximate spike cluster solution and consider the linearised operator around this ansatz. We compute the remainder when this ansatz is plugged into the Gierer–Meinhardt system. Then we introduce the linearised operator around this ansatz. Key results for Liapunov–Schmidt reduction will proved in appendix A.

Step 2.: In appendix A key results for the method of Liapunov–Schmidt reduction are proved. It is shown that this linearised operator as well as the conjugate operator are uniformly invertible modulo the kernel and cokernel consisting of the translation modes. Then it is shown that the fully nonlinear system has a solution modulo the kernel and cokernel. This implies that the existence problem can be reduced to a finite-dimensional problem.
Step 3.: In Sect. 4 the reduced problem is solved. $\square$

Next we state our second result which concerns the stability of the *k*-spike cluster steady states given in Theorem 1.1. Whereas the existence result is valid for any number $k\ge 2$ of spikes, the stability or instability of spike clusters depends on *k*.

### Theorem 1.2

(Stability of *k*-spike clusters). For $\max \left( \frac{\varepsilon }{\sqrt{D}},D\log \frac{\sqrt{D}}{\varepsilon }\right)$ sufficiently small, let $A_{\varepsilon },H_{\varepsilon }$ be the *k*-spike cluster steady state given in Theorem 1.1. Then there exists $\tau _0>0$ which is independent of $\varepsilon$ and *D* but may depend on *k* such that the *k*-spike cluster steady state $(A_{\varepsilon },H_{\varepsilon })$ is linearly stable for $2\le {k\le 3}$ and unstable for $k\ge 5$ provided that $0\le \tau <\tau _0$.

The main existence and stability results can be extended to a cluster with *k* spikes located on a regular polygon plus an extra spike in its centre.

### Proposition 1.3

For any $k\ge 2$ there also exists a $(k+1)$-spike cluster steady state similar to the one given in Theorem 1.1 but with an extra spike in the centre of the regular polygon. This $(k+1)$-spike cluster is linearly stable if $k\le 5$ provided that $0\le \tau <\tau _0$ for some $\tau _0>0$.

### Remark 4

These results suggest that placing a spike in the centre of a polygon can stabilise the spike cluster in the following sense: by putting a spike in the centre of the polygon it is possible to get a stable polygonal spike cluster containing six spikes but without a centre the number of spikes for a stable cluster cannot be five or more.

### Remark 5

The stability of a spike cluster with four spikes on a regular polygon remains open. The stability problem in this case requires further expansion of an eigenvalue which is zero in the two leading orders. One mode which has to be resolved is that of two opposite spikes moving towards the centre and the other two spikes moving away from the centre without changing the distance between neighbours.

### Remark 6

One instability of a spike cluster with five or more spikes on a regular polygon comes from a mode such that the spikes tend to move away from their original positions on a circle, some to the inside and some to the outside, since this increases the distances between neighbouring spikes.

### Remark 7

The stability of a spike cluster with spikes on a regular polygon with six vertices plus its centre remains open. The stability problem in this case requires some new analysis since each spike now has three neighbours which have the same smallest distance (two spikes on the circle plus the spike in the centre).

### Remark 8

One instability of a spike cluster with seven or more spikes on a regular polygon with centre comes from a mode such that the spikes tend to move away from their original positions on a circle, some to the inside and some to the outside, since this increases the distances between neighbouring spikes.

*Key steps in the proof of Theorem* 1.2:

### Proof

To study the stability of a *k*-spike cluster we have to study large eigenvalues of order *O*(1) and small eigenvalues of order *o*(1) separately.

Step 1. In Sect. 5 we consider eigenvalues of order *O*(1). We show, using the results of Sect. 2, that they all have negative real part. This part applies to all $k\ge 2$.
Step 2. In appendix B we consider small eigenvalues and reduce the stability problem to a finite-dimensional problem. This part applies to all $k\ge 2$.
Step 3. In Sect. 6 we study this finite-dimensional problem. The computations depend on the number *k* of spikes in an essential way. At the end of Sect. 6 the spectral properties for different values of *k* are studied separately. $\square$

*Key steps in the proof of Proposition* 1.3:

### Proof

Step 1. The same as Step 1 in the proof of Theorem 1.2.

Step 2.: The same as Step 2 in the proof of Theorem 1.2.
Step 3.: In Sect. 7 we study the finite-dimensional problem. The computations depend on the number *k* of spikes in an essential way. At the end of Sect. 7 the spectral properties for different values of *k* are studied separately. $\square$

**Fig. 1:**
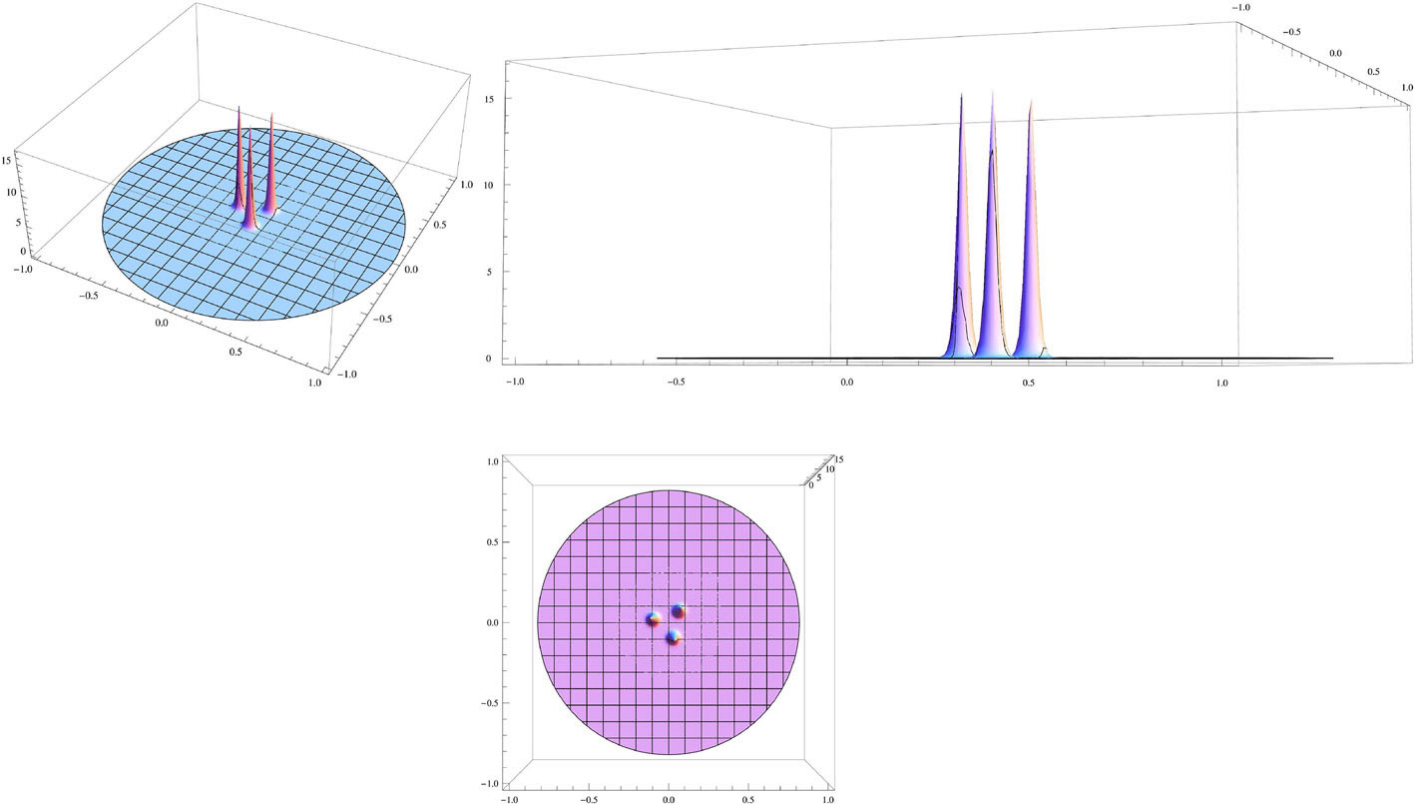
Clustered spiky steady state of (1.2) for $\varepsilon ^2=0.001,\,D=0.01,\,\mu (|y|)=1+5 |y|^2$. Shown is a 3-spike cluster consisting of 3 spikes on a regular polygon. The activator *A* is displayed in two three-dimensional surface plots from different perspectives in the *top two graphs* and its projection to the domain plane is shown in the *bottom graph*

**Fig. 2:**
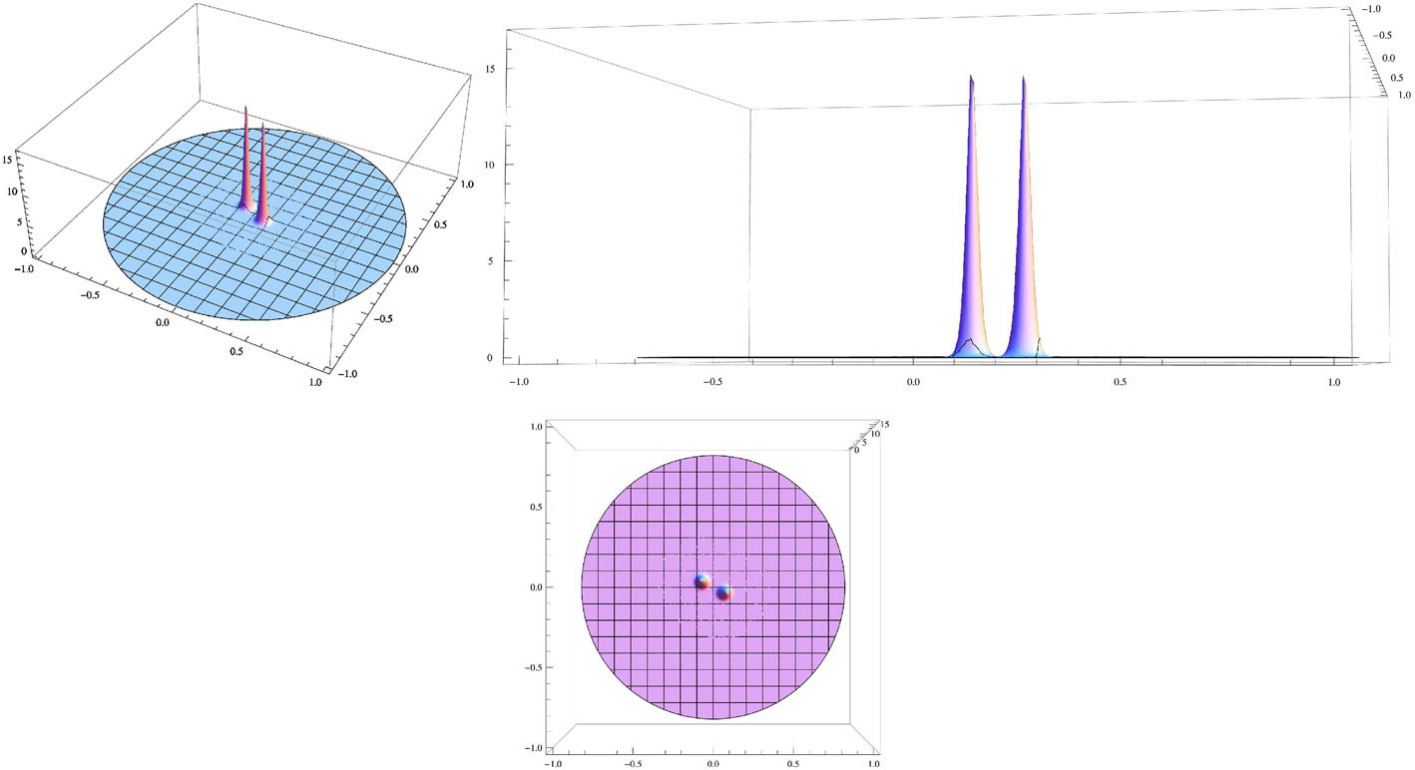
Clustered spiky steady state of (1.2) for $\varepsilon ^2=0.001,\,D=0.01,\,\mu (|y|)=1+5 |y|^2$. Shown is a 2-spike cluster consisting of 2 spikes on a regular polygon. The activator *A* is displayed in two three-dimensional surface plots from different perspectives in the *top two graphs* and its projection to the domain plane is shown in the *bottom graph*

**Fig. 3:**
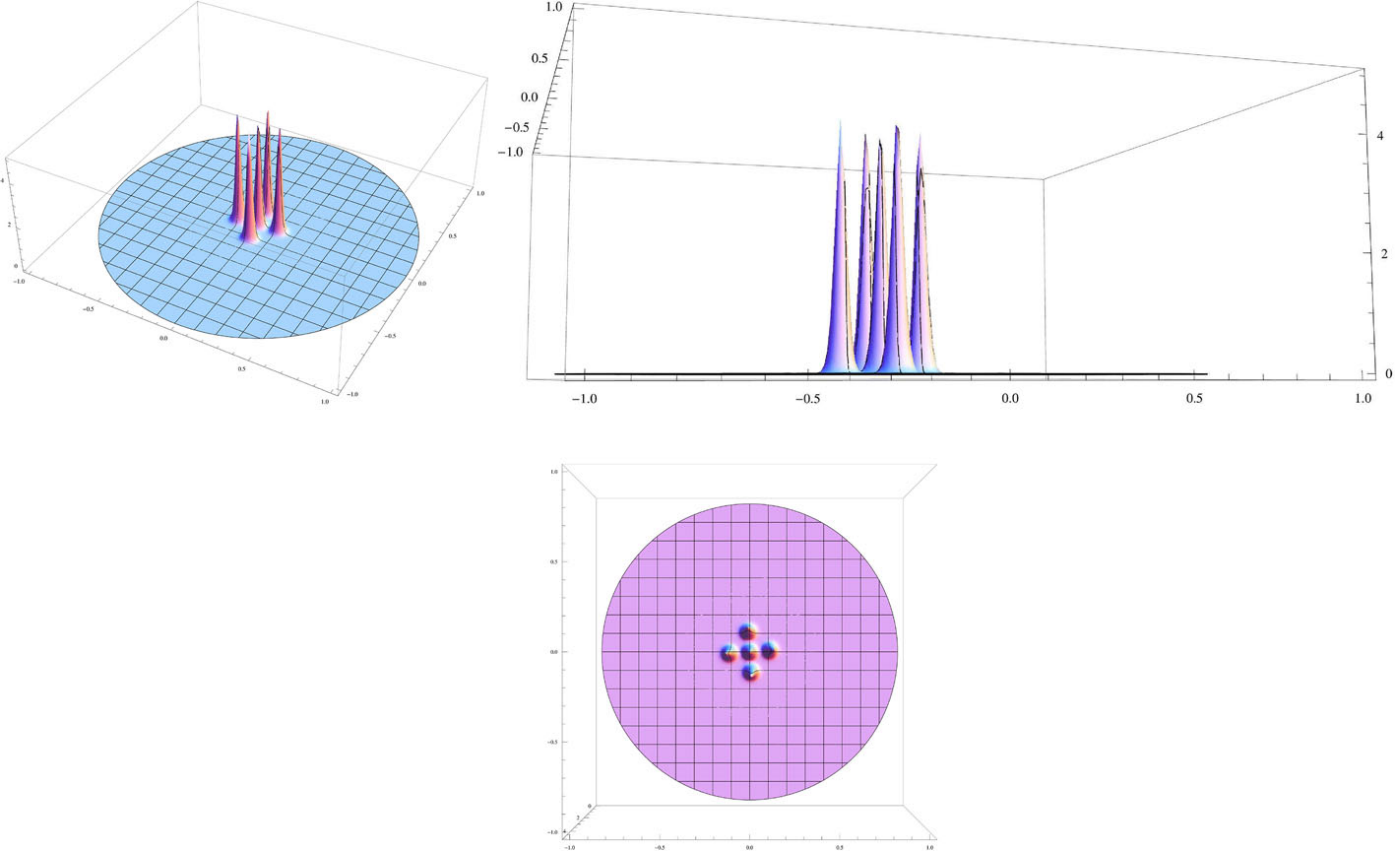
Clustered spiky steady state of (1.2) for $\varepsilon ^2=0.0001,\,D=0.002,\,\mu (|y|)=1+5 |y|^2$. Because smaller diffusivities are chosen, we now get more spikes than in Figs. 1 and 2. Shown is a $(4+1)$-spike cluster consisting of 4 spikes on a regular polygon plus a spike in the centre. The activator *A* is displayed in two three-dimensional surface plots from different perspectives in the *top two graphs* and its projection to the domain plane is shown in the *bottom graph*

We confirm and illustrate the main results by a few numerical computations (Figs. 1,2,3).

This paper is organised as follows. In Sect. 2 we present some preliminaries on the spectral properties of the nonlocal linear operators which will appear in the existence proof and in the stability proof for the analysis of large eigenvalues of order *O*(1). We study the existence of a *k*-spike cluster solution to (1.2) in Sect. 3 and appendix A (Liapunov–Schmidt reduction) and Sect. 4 (solving the reduced problem); in appendix A we prove some key results for Liapunov–Schmidt reduction which are needed in Sect. 3. In Sect. 5 we rigourously study the large eigenvalues of order *O*(1) for the linearised problem around the steady state spike cluster. The small eigenvalues of order *o*(1) are investigated in appendix C (general theory) and Sect. 6 (explicit computation of small eigenvalues which decide the stability of spike clusters). In Sect. 7 we sketch how the approach can be adapted to show existence and stability of a cluster for which the spikes are located at the vertices of a regular polygon with centre.

Note that in Sect. 6 the number *k* of spikes plays an explicit role and different values of *k* are considered separately. In the same way, in Sect. 7 the number $k+1$ of spikes plays a role and the treatment depends on the value of *k*. In particular, the different cases of *k* are studied towards end of Sects. 6 and 7, respectively. The other sections of the paper apply to all values of $k\ge 2$ in the same way.

Throughout the paper, by *C*, *c* we denote generic constants which may change from line to line. Further, *h*.*o*.*t*. stands for higher order terms.

## Preliminaries: spectral properties of some eigenvalue problems

In this section we shall provide some preliminaries which will be needed for the existence and stability proofs.

Let *w* be the ground state solution given in (1.3), i.e, the unique solution of the problem

2.1 $$\begin{aligned} {\left\{ \begin{array}{ll} \Delta w-w+w^2=0,~y\in \mathbb {R}^2,\quad w>0,\\ w(0)=\max _{x\in \mathbb {R}^2}w(x),~w(x)\rightarrow 0~\mathrm {as}~|x|\rightarrow 0. \end{array}\right. } \end{aligned}$$

Let

2.2 $$\begin{aligned} L_0\phi =\Delta \phi -\phi +2w\phi ,~\phi \in H^2(\mathbb {R}^2), \end{aligned}$$

where

$$\begin{aligned} H^2(\mathbb {R}^2)=\left\{ u\in L^2(\mathbb {R}^2)\,:\, \frac{\partial u}{\partial x_i}\in L^2(\mathbb {R}^2),\, \frac{\partial ^2 u}{\partial x_i \partial x_j}\in L^2(\mathbb {R}^2),\,i,j=1,2\right\} \end{aligned}$$

and $L^2(\mathbb {R}^2)$ is the space of all square integrable functions defined on $\mathbb {R}^2$.

We first recall the following well known result:

### Lemma 2.1

The eigenvalue problem

2.3 $$\begin{aligned} L_0\phi =\lambda \phi ,~\phi \in H^2(\mathbb {R}^2), \end{aligned}$$

admits the following set of eigenvalues

2.4 $$\begin{aligned} \lambda _1>0,~\lambda _2=\lambda _3=0,~\lambda _4<0,{\ldots }. \end{aligned}$$

The eigenfunction $\Phi _0$ corresponding to $\lambda _1$ can be made positive and radially symmetric, the space of eigenfunctions corresponding to the eigenvalue 0 is

$$\begin{aligned} K_0:=\mathrm {span}\left\{ \frac{\partial w}{\partial x_j},j=1,2\right\} . \end{aligned}$$

### Proof

This lemma follows from [10, Theorem 2.1] and [14, Lemma C]. $\square$

Next we consider the following nonlocal eigenvalue problem:

2.5 $$\begin{aligned} \Delta \phi -\phi +2w\phi -2\frac{\int _{\mathbb {R}^2}w\phi \,\mathrm {d}x}{\int _{\mathbb {R}^2}w^2\,\mathrm {d}x}w^2=\alpha _0\phi ,~\phi \in H^2(\mathbb {R}^2). \end{aligned}$$

Problem (2.5) plays a key role in the study of large eigenvalues (See Sect. 5 below). For problem (2.5), we have the following theorem due to [19, Theorem 1.4].

### Theorem 2.2

Let $\alpha _0\ne 0$ be an eigenvalue of the problem (2.5). Then we have $\mathfrak {R}(\alpha _0)\le -c_1$ for some $c_1>0$.

We shall also consider the following system of nonlocal eigenvalue problems:

2.6 $$\begin{aligned} L\Phi :=\Delta \Phi -\Phi +2w\Phi -2\frac{\int _{\mathbb {R}^2}w\Phi \,\mathrm {d}x}{\int _{\mathbb {R}^2}w^2\,\mathrm {d}x}w^2, \end{aligned}$$

where

$$\begin{aligned} \Phi :=\left( \phi _1,\phi _2,\ldots ,\phi _k\right) ^T \in (H^2(\mathbb {R}^2))^k. \end{aligned}$$

Then the conjugate operator of *L* under the scalar product in $L^2(\mathbb {R}^2)$ is given by

2.7 $$\begin{aligned} L^*\Phi =\Delta \Phi -\Phi +2w\Phi -2\frac{\int _{\mathbb {R}^2}w^2\Phi \,\mathrm {d}x}{\int _{\mathbb {R}^2}w^2\,\mathrm {d}x}w. \end{aligned}$$

We have the following result:

### Lemma 2.3

We have

$$\begin{aligned} Ker(L)=K_0\oplus K_0\oplus \cdots \oplus K_0, \end{aligned}$$

and

$$\begin{aligned} Ker(L^*)=K_0\oplus K_0\oplus \cdots \oplus K_0. \end{aligned}$$

### Proof

The system (2.6) is in diagonal form. Suppose

$$\begin{aligned} L\Phi =0. \end{aligned}$$

For $i=1,2,\ldots ,k$ the *i*-th equation of (2.6) is given by

2.8 $$\begin{aligned} \Delta \phi _i-\phi _i+2w\phi _i-2\frac{\int _{\mathbb {R}^2}w\phi _i\,\mathrm {d}x}{\int _{\mathbb {R}^2}w^2\,\mathrm {d}x}w^2=0. \end{aligned}$$

We claim that (2.8) admits only the solution $\frac{\partial w}{\partial x_i},i=1,2$. Indeed, we note that $\phi _i'=\frac{\int _{\mathbb {R}^2}w\phi _i\,\mathrm {d}x}{\int _{\mathbb {R}^2}w^2\,\mathrm {d}x}w$ satisfies that

$$\begin{aligned} \Delta \phi _i'-\phi _i'+2w\phi _i'=\frac{\int _{\mathbb {R}^2}w\phi _i\,\mathrm {d}x}{\int _{\mathbb {R}^2}w^2\,\mathrm {d}x}w^2. \end{aligned}$$

As a result, $\phi _i-{2}\phi _i'$ satisfies

$$\begin{aligned} \Delta \left( \phi _i-2\phi _i'\right) -\left( \phi _i-2\phi _i'\right) +2w\left( \phi _i-2\phi _i'\right) =0, \end{aligned}$$

and we get

2.9 $$\begin{aligned} \phi _i-2\phi _i'=c_1\frac{\partial w}{\partial x_1}+c_2\frac{\partial w}{\partial x_2} \end{aligned}$$

by Lemma 2.1. Multiplying by *w* on both sides of (2.9) and integrating, we have $\int _{\mathbb {R}^2}w\phi _i\,\mathrm {d}x=0$. Hence, $\phi _i$ is the solution to

$$\begin{aligned} \Delta \phi -\phi +2w\phi =0, \end{aligned}$$

and we get the first conclusion of Lemma 2.3. To prove the second statement, we proceed in a similar way for $L^*$, and the *i*-th equation of (2.7) is given as

2.10 $$\begin{aligned} \Delta \phi _i-\phi _i+2w\phi _i-2\frac{\int _{\mathbb {R}^2}w^2\phi _i\,\mathrm {d}x}{\int _{\mathbb {R}^2}w^2\,\mathrm {d}x}w=0. \end{aligned}$$

Multiplying (2.10) by *w* and integrating, we obtain $\int _{\mathbb {R}^2}w^2\phi _i\,\mathrm {d}x=0$. Then we have

$$\begin{aligned} \Delta \phi _i-\phi _i+2w\phi _i=0. \end{aligned}$$

By Lemma 2.1 again, we get the second conclusion and the proof is finished. $\square$

By the result of Lemma 2.3, we have

### Lemma 2.4

The operator

$$\begin{aligned} L:(H^2(\mathbb {R}^2))^k\rightarrow {(L^2(\mathbb {R}^2))^k}, L\Phi =\Delta \Phi -\Phi +2w\Phi -2\frac{\int _{\mathbb {R}^2}w\Phi \,\mathrm {d}x}{\int _{\mathbb {R}^2}w^2\,\mathrm {d}x}w^2, \end{aligned}$$

is invertible if it is restricted as follows:

$$\begin{aligned} L:\left( K_0\oplus \cdots \oplus K_0\right) ^{\perp }\cap (H^2(\mathbb {R}^2))^k\rightarrow \left( K_0\oplus \cdots \oplus K_0\right) ^{\perp }\cap (L^2(\mathbb {R}^2))^k. \end{aligned}$$

Moreover, $L^{-1}$ is bounded.

### Proof

This results follows from the Fredholm Alternative and Lemma 2.3. $\square$

Next we study the eigenvalue problem for *L* : 

2.11 $$\begin{aligned} L\Phi =\alpha \Phi . \end{aligned}$$

We have

### Lemma 2.5

For any nonzero eigenvalue $\alpha$ of (2.11) we have $\mathfrak {R}(\alpha )\le -c<0$.

### Proof

Let $(\Phi ,\alpha )$ satisfy the system (2.11). Suppose $\mathfrak {R}(\alpha )\ge 0$ and $\alpha \ne 0$. The *i*-th equation of (2.11) becomes

$$\begin{aligned} \Delta \phi _i-\phi _i+2w\phi _i-2\frac{\int _{\mathbb {R}^2}w\phi _i\,\mathrm {d}x}{\int _{\mathbb {R}^2}w^2\,\mathrm {d}x}\phi _i=\alpha \phi _i. \end{aligned}$$

By Theorem 2.2, we conclude that

$$\begin{aligned} \mathfrak {R}(\alpha )\le -c<0. \end{aligned}$$

$$\square $$

It is interesting to observe that for spike clusters the operator in the nonlocal eigenvalue problem (2.6) and its conjugate operator (2.7) take diagonal form. Thus they can be studied very easily by considering the scalar nonlocal eigenvalue problem which arises from the study of a single spike. In other words, for a spike cluster in the nonlocal eigenvalue problem each spike “feels” only itself and not the other spikes.

## Existence I: reduction to finite dimensions

In this section, we shall reduce the existence problem to a finite-dimensional problem. In the first step, we choose a good approximation to an equilibrium state. Then we shall use Liapunov–Schmidt reduction to reduce the original problem to a finite-dimensional one. Some key results for Liapunov–Schmidt reduction are proved in appendix A. In the next section, we solve the reduced problem.

First of all, let us set the candidate points for the location of spikes to the activator. Let $Q_{\varepsilon }$ denote the set of the vertex points of the regular *k*-polygon:

3.1 $$\begin{aligned} Q_{\varepsilon }=\left\{ \mathbf{q}=(q_1,\ldots ,q_k)\mid q_i=\left( 2R_{\varepsilon }\cos \frac{2(i-1)\pi }{k},2R_{\varepsilon }\sin \frac{2(i-1)\pi }{k}\right) \right\} , \end{aligned}$$

where $R_{\varepsilon }$ is chosen such that

3.2 $$\begin{aligned} \frac{1}{C}\frac{\sqrt{D}}{\varepsilon }\log \left( \frac{1}{D\log \frac{\sqrt{D}}{\varepsilon }}\right) \le R_{\varepsilon }\le C\frac{\sqrt{D}}{\varepsilon }\log \left( \frac{1}{D\log \frac{\sqrt{D}}{\varepsilon }}\right) \end{aligned}$$

for some constant $C>1$ independent of $\varepsilon$ and *D*. If $\max \left( \frac{\varepsilon }{\sqrt{D}},D\log \frac{\sqrt{D}}{\varepsilon }\right) \rightarrow 0$, then we can see that $\varepsilon R_{\varepsilon }\rightarrow 0$ and $\frac{\sqrt{D}}{\varepsilon R_{\varepsilon }}\rightarrow 0$.

Recall that we want to solve (1.4) which is given by

$$\begin{aligned} {{\left\{ \begin{array}{ll} \Delta A-\mu (|\varepsilon x|)A+\frac{A^2}{H}=0,\quad &{}\mathrm {in}~B_{R/\varepsilon },\\ \Delta H-\sigma ^2H+\xi A^2=0,\quad &{}\mathrm {in}~B_{R/\varepsilon },\\ \frac{\partial A}{\partial \nu }=\frac{\partial H}{\partial \nu }=0, \quad &{}\text{ on }~\partial B_{R/\varepsilon }, \end{array}\right. }} \end{aligned}$$

where $$\sigma ^2=\frac{\varepsilon ^2}{D}$$.

Next we introduce the cut-off function $\chi _{\varepsilon ,q_j}(x)=\chi (\frac{x-q_j}{R_{\varepsilon }\sin \frac{\pi }{k}})$, where $\chi$ is a function which satisfies

3.3 $$\begin{aligned} \chi (x)={\left\{ \begin{array}{ll}1,~|x|\le \frac{1}{2},\\ 0,~|x|>1,\end{array}\right. }~\chi \in C_0^{\infty }(\mathbb {R}^2). \end{aligned}$$

The gap between $\frac{1}{2}$ and 1 is to be filled with an arbitrary function that bridges the two parts infinitely smoothly.

Let $(q_1,\ldots ,q_k)$ be defined as in (3.1) and we set

$$\begin{aligned} W=\sum _{j=1}^k\chi _{\varepsilon ,q_j}(x)w(x-q_j). \end{aligned}$$

Let *G*(*x*, *z*) be the Green function given by

$$\begin{aligned} {\left\{ \begin{array}{ll} \Delta _x G(x,z)-G(x,z)+\delta _z(x)=0~&{}\mathrm {in}~B_{R/\sqrt{D}},\\ \frac{\partial G(x,z)}{\partial \nu }=0~&{}\mathrm {on}~\partial B_{R/\sqrt{D}}. \end{array}\right. } \end{aligned}$$

We have

1. If $0<|x-z|\ll 1$, we have
  3.4 $$\begin{aligned} G(x,z)=\frac{1}{2\pi }\log \frac{1}{|x-z|}+H(x,z), \end{aligned}$$
  where *H*(*x*, *z*) is a continuous function and $\nabla _x H(x,z)|_{x=z}=0$.
2. If $1\ll |x-z|\ll R$, we have
  3.5 $$\begin{aligned} G(x,z)=C|x-z|^{-\frac{1}{2}}e^{-|x-z|}\left( 1+o(1)\right) ,~|\nabla _x G(x,z)|=G(x,z)(1+o(1)) \end{aligned}$$
  for some generic constant *C*.

We write

3.6 $$\begin{aligned} \xi ^{-1}=\int _{B_{R/\varepsilon }}G(\sigma q_1,\sigma z)\left( \sum _{j=1}^k\chi (\varepsilon z)w(z-q_j)\right) ^2\mathrm {d}z= \frac{1}{2\pi }\log \frac{1}{\sigma }\left( {\int _{\mathbb {R}^2}w^2\,\mathrm {d}z}+o(1)\right) . \end{aligned}$$

For a function $u\in H^2(B_{R/\varepsilon })$, let *T*[*u*] be the unique solution in $H^2_N(B_{R/\varepsilon })$ of the following problem:

3.7 $$\begin{aligned} \Delta T[u]-\sigma ^2 T[u]+\xi u^2=0~\mathrm {in}~B_{R/\varepsilon }, \end{aligned}$$

where

$$\begin{aligned} H^2_N(B_{R/\varepsilon })=\left\{ u\in H^2(B_{R/\varepsilon })\,|\, \frac{\partial u}{\partial \nu }=0 \ \text{ on } \partial B_{R/\varepsilon }\right\} . \end{aligned}$$

Written differently, we have

3.8 $$\begin{aligned} T[u](x)=\xi \int _{B_{R/\varepsilon }}G_{\sigma }(x,z)u^2(z)\,\mathrm {d}y, \end{aligned}$$

where $G_{\sigma }(x,z)$ is the Green function which satisfies $(\Delta -\sigma ^2)G_{\sigma }(x,z)+\delta _z(x)=0$ in $B_{R/\varepsilon }$ with Neumann boundary condition. Note that

3.9 $$\begin{aligned} G_\sigma (x,z)=G(\sigma x,\sigma z)\quad \text{ for } x,z\in B_{R/\varepsilon }. \end{aligned}$$

System (1.4) is equivalent to the following equation in operator form:

3.10 $$\begin{aligned} S_{\varepsilon }(u,v)= \left( \begin{matrix} S_1(A,H)\\ S_2(A,H)\end{matrix}\right) =0,~ H^2_N(B_{R/\varepsilon })\times H^2_N(B_{R/\varepsilon })\rightarrow L^2(B_{R/\varepsilon })\times L^2(B_{R/\varepsilon }), \end{aligned}$$

where

$$\begin{aligned} S_1(A,H)&=\Delta A-\mu (|\varepsilon x|)A+\frac{A^2}{H}:~H^2_N(B_{R/\varepsilon })\times H^2_N(B_{R/\varepsilon })\rightarrow L^2(B_{R/\varepsilon }),\\ S_2(A,H)&=\Delta H-\sigma ^2H+\xi A^2:~H^2_N(B_{R/\varepsilon })\times H^2_N(B_{R/\varepsilon })\rightarrow L^2(B_{R/\varepsilon }). \end{aligned}$$

For Eq. (1.4), we choose our approximate solution as follows,

3.11 $$\begin{aligned} A_{\varepsilon ,\mathbf {q}}=W,~H_{\varepsilon ,\mathbf {q}}=T[W]. \end{aligned}$$

Note that $H_{\varepsilon ,\mathbf {q}}$ satisfies

$$\begin{aligned} 0=\Delta H_{\varepsilon ,\mathbf {q}}-\sigma ^2H_{\varepsilon ,\mathbf {q}}+\xi A_{\varepsilon ,\mathbf {q}}^2=\Delta H_{\varepsilon ,\mathbf {q}}-\sigma ^2H_{\varepsilon ,\mathbf {q}}+\xi \sum _{j=1}^kw(x-q_j)^2+h.o.t.. \end{aligned}$$

Further, by our choice of $\xi$ in (3.6), it is easy to see that $H_{\varepsilon ,\mathbf {q}}(q_i)=1,~i=1,\ldots ,k$. We insert our ansatz (3.11) into (1.4) and calculate

3.12 $$\begin{aligned} S_2(A_{\varepsilon ,\mathbf {q}},H_{\varepsilon ,\mathbf {q}})=0, \end{aligned}$$

and

3.13 $$\begin{aligned} S_1(A_{\varepsilon ,\mathbf {q}},H_{\varepsilon ,\mathbf {q}})&=\Delta A_{\varepsilon ,\mathbf {q}}-\mu (|\varepsilon x|)A_{\varepsilon ,\mathbf {q}}+\frac{A_{\varepsilon ,\mathbf {q}}^2}{H_{\varepsilon ,\mathbf {q}}}\nonumber \\&=\sum _{j=1}^k\Big [\Delta w(x-q_j)-\mu (|\varepsilon x|)w(x-q_j)\Big ]+\sum _{j=1}^kw^2(x-q_j)H_{\varepsilon ,\mathbf {q}}^{-1}+h.o.t.\nonumber \\&=\sum _{j=1}^k\big (1-\mu (|\varepsilon x|)\big )w(x-q_j)+\sum _{j=1}^kw^2(x-q_j)\big (H_{\varepsilon ,\mathbf {q}}^{-1}-1\big )+h.o.t. \end{aligned}$$

On the other hand, we calculate for $j=1,\ldots ,k$ and $x=q_j+ z$ with $q_j=(q_{j,1},q_{j,2})$ in the range $|\sigma z|<\delta$ for $\delta >0$ small enough:

3.14 $$\begin{aligned} H_{\varepsilon ,\mathbf {q}}(q_j+ z)-1=~&\xi \int _{B_{R/\varepsilon }}\Big (G_{\sigma } (q_j+z,t)-G_{\sigma }(q_j,t)\Big )A^2_{\varepsilon ,\mathbf {q}}\,\mathrm {d}t\nonumber \\ =~&\xi \int _{B_{R/\varepsilon }}\Big (G_{\sigma }(q_j+z,t)-G_{\sigma } (q_j,t)\Big )w(t-q_j)^2\,\mathrm {d}t\nonumber \\&+\xi \int _{B_{R/\varepsilon }}\Big (G_{\sigma }(q_j+z,t)-G_{\sigma } (q_j,t)\Big )\sum _{l,\,l\ne j}w(t-q_l)^2\,\mathrm {d}t\nonumber \\&+O\left( e^{-2R_{\varepsilon }\sin \left( \frac{\pi }{k}\right) }\right) \nonumber \\ =~&\xi \int _{\mathbb {R}^2}\frac{1}{2\pi }\log \frac{|t|}{|z-t|}w^2(t)\,\mathrm {d}t +\xi \left( \sum _{l=1}^2\frac{\partial F(\mathbf {q})}{\partial q_{j,l}} \sigma z_l\int _{\mathbb {R}^2}w^2(t)\,\mathrm {d}t\right) \nonumber \\&+\xi \sum _{l,m=1}^2\frac{\partial ^2F(\mathbf {q})}{\partial q_{j,l} \partial q_{j,m}}\sigma ^2z_lz_m\int _{\mathbb {R}^2}w^2(t)\,\mathrm {d}t +O\left( e^{-2R_{\varepsilon }\sin \left( \frac{\pi }{k}\right) }\right) \nonumber \\&+O\left( \sigma ^3|z|^2+\sigma ^2R_{\sigma }^{-\frac{1}{2}}e^{-R_{\sigma }}|z|\right) , \end{aligned}$$

where $$R_{\sigma }=2\sigma R_{\varepsilon }\sin \left( \frac{\pi }{k}\right) $$ and

3.15 $$\begin{aligned} F(\mathbf {q})=\sum _{i=1}^kH(\sigma q_i,\sigma q_i)+\sum _{i,j, i\ne j}G(\sigma q_i,\sigma q_j). \end{aligned}$$

Note that the error term in (3.14) consists of three parts. The first part is very small and comes from the difference between $A_{\varepsilon ,\mathbf {q}}$ and *w* due to the decay of the activator component between spikes and near the boundary. The second part estimates higher order terms in the expansion of $F(\mathbf {q})$ around $\mathbf {q}$. The third part estimates the smallness of the contribution of non-neighbouring spikes in $F(\mathbf {q})$. The same three types of error terms appear repeatedly throughout the paper.

Substituting (3.14) into (3.13), we have the following key estimate,

### Lemma 3.1

For $x=q_j+z,~|\sigma z|<\delta$ and $\delta >0$ small enough, we have

3.16 $$\begin{aligned} S_1(A_{\varepsilon ,\mathbf {q}},H_{\varepsilon ,\mathbf {q}})=S_{1,1}+S_{1,2}, \end{aligned}$$

where

3.17 $$\begin{aligned} S_{1,1}(z)&=\xi H_{\varepsilon ,\mathbf {q}}(q_j)^{-2}\left( \int _{\mathbb {R}^2}w^2(t) \,\mathrm {d}t\right) w^2(z)\Big (\sigma z\cdot \nabla _{q_j}F(\mathbf {q})\nonumber \\&\quad +\sigma ^2\sum _{l,m=1}^2 z_lz_m\frac{\partial ^2F(\mathbf q)}{\partial q_{j,l}\partial q_{j,m}}+h.o.t.\Big )\nonumber \\&\quad +\varepsilon \sum _{l=1}^2z_l\left( \mu ''(0)+\frac{1}{2}\mu '''(0)\varepsilon |q_j| +O(\varepsilon ^2|q_j|^2)\right) \varepsilon q_{j,l}\sum _{i=1}^kw(x-q_i)\nonumber \\&\quad +O\left( \sigma ^3|z|^2+\sigma ^2R_{\sigma }^{-\frac{1}{2}}e^{-R_{\sigma }}|z|+e^{-2R_{\varepsilon } \sin \left( \frac{\pi }{k}\right) }\right) , \end{aligned}$$

and

3.18 $$\begin{aligned} S_{1,2}(z)=\xi w^2(z)R(|z|)+\varepsilon ^2R_{\varepsilon }^2w(z), \end{aligned}$$

where *R*(|*z*|) is a radially symmetric function with the property that

$$\begin{aligned} R(|z|)=O(\log (1+|z|)). \end{aligned}$$

Further, $$S_1(A_{\varepsilon ,\mathbf {q}},H_{\varepsilon ,\mathbf {q}})=e^{-\frac{\delta }{\sigma }}$$ for $$|x-q_j|\ge \frac{\delta }{\sigma },~j=1,2,\ldots ,k$$.

The above estimates will be very important in the following calculations, where (3.10) is solved modulo kernel and cokernel.

Now we study the linearised operator defined by

$$\begin{aligned}&{\tilde{L}}_{\varepsilon ,\mathbf {q}}:=S_{\varepsilon ,\mathbf {q}}'\left( \begin{matrix}A_{\varepsilon ,\mathbf {q}}\\ H_{\varepsilon ,\mathbf {q}}\end{matrix}\right) ,\\&{\tilde{L}}_{\varepsilon ,\mathbf {q}}:H^2_N(B_{R/\varepsilon })\times H^2_N (B_{R/\varepsilon })\rightarrow L^2(B_{R/\varepsilon })\times L^2(B_{R/\varepsilon }). \end{aligned}$$

Set

$$\begin{aligned} K_{\varepsilon ,\mathbf {q}}:=\mathrm {span}\left\{ \frac{\partial A_{\varepsilon ,\mathbf {q}}}{\partial q_{j,l}}\mid j=1,2,\ldots ,k,l=1,2\right\} \subset H^2_N(B_{R/\varepsilon }), \end{aligned}$$

and

$$\begin{aligned} C_{\varepsilon ,\mathbf {q}}:=\mathrm {span}\left\{ \frac{\partial A_{\varepsilon ,\mathbf {q}}}{\partial q_{j,l}}\mid j=1,2,\ldots ,k,l=1,2\right\} \subset L^2(B_{R/\varepsilon }). \end{aligned}$$

The operator ${\tilde{L}}_{\varepsilon ,\mathbf {q}}$ is not uniformly invertible in $\sigma$ and $D\log \frac{1}{\sigma }$ due to its approximate kernel

3.19 $$\begin{aligned} \mathcal {K}_{\varepsilon ,\mathbf {q}}:=K_{\varepsilon ,\mathbf {q}}\oplus \{0\}\subset H^2_N(B_{R/\varepsilon })\times H^2_N(B_{R/\varepsilon }). \end{aligned}$$

and approximate cokernel

3.20 $$\begin{aligned} \mathcal {C}_{\varepsilon ,\mathbf {q}}:=C_{\varepsilon ,\mathbf {q}}\oplus \{0\}\subset L^2(B_{R/\varepsilon })\times L^2(B_{R/\varepsilon }). \end{aligned}$$

Then we define

3.21 $$\begin{aligned} \mathcal {K}_{\varepsilon ,\mathbf {q}}^{\perp }&:=K_{\varepsilon ,\mathbf {q}}^{\perp }\oplus H^2_N(B_{R/\varepsilon })\subset H^2_N(B_{R/\varepsilon })\times H^2_N(B_{R/\varepsilon }),\end{aligned}$$

3.22 $$\begin{aligned} \mathcal {C}_{\varepsilon ,\mathbf {q}}^{\perp }&:=C_{\varepsilon ,\mathbf {q}}^{\perp }\oplus L^2(B_{R/\varepsilon })\subset L^2(B_{R/\varepsilon })\times L^2(B_{R/\varepsilon }), \end{aligned}$$

where $\mathcal {C}_{\varepsilon ,\mathbf {q}}^{\perp }$ and $\mathcal {K}_{\varepsilon ,\mathbf {q}}^{\perp }$ denote the orthogonal complement with the scalar product of $L^2(B_{R/\varepsilon })$ in the spaces $H^2_N(B_{R/\varepsilon })$ and $L^2(B_{R/\varepsilon })$, respectively.

Let $\pi _{\varepsilon ,\mathbf {q}}$ denote the projection in $L^2(B_{R/\varepsilon })\times L^2(B_{R/\varepsilon })$ onto $\mathcal {C}_{\varepsilon ,\mathbf {q}}^{\bot }$, where the second component of the projection is the identity map. We are going to show that the equation

$$\begin{aligned} \pi _{\varepsilon ,\mathbf {q}}\circ S_{\varepsilon ,\mathbf {q}}\left( \begin{matrix} A_{\varepsilon ,\mathbf {q}}+\phi _{\varepsilon ,\mathbf {q}}\\ H_{\varepsilon ,\mathbf {q}}+\psi _{\varepsilon ,\mathbf {q}} \end{matrix}\right) =0 \end{aligned}$$

has a unique solution $$\Sigma _{\varepsilon ,\mathbf {q}}=\left( \begin{matrix} \phi _{\varepsilon ,\mathbf {q}}\\ \psi _{\varepsilon ,\mathbf {q}} \end{matrix}\right) \in \mathcal {K}_{\varepsilon ,\mathbf {q}}^{\perp }$$ if $\max \left( \frac{\varepsilon }{\sqrt{D}},D\log \frac{\sqrt{D}}{\varepsilon }\right)$ is small enough (Liapunov–Schmidt reduction).

Set

3.23 $$\begin{aligned} \mathcal {L}_{\varepsilon ,\mathbf {q}}=\pi _{\varepsilon ,\mathbf {q}}\circ {\tilde{L}}_{\varepsilon ,\mathbf {q}}: \mathcal {K}_{\varepsilon ,\mathbf {q}}^{\perp }\rightarrow \mathcal {C}_{\varepsilon ,\mathbf {q}}^{\perp }. \end{aligned}$$

In appendix A we will show the following key results for Liapunov–Schmidt reduction:

1. The linear operator $\mathcal {L}_{\varepsilon ,\mathbf {q}}$ is uniformly invertible.
2. There exists $$\Sigma _{\varepsilon ,\mathbf {q}}=\left( \begin{matrix} \phi _{\varepsilon ,\mathbf {q}}\\ \psi _{\varepsilon ,\mathbf {q}} \end{matrix} \right) \in \mathcal {K}_{\varepsilon ,\mathbf {q}}^{\perp }$$.

Then, in the next section, we will solve the reduced problem and determine the point $\mathbf {q}\in Q_{\varepsilon }$.

## Existence II: the reduced problem

In this section, we solve the reduced problem and complete the proof of Theorem 1.1.

By Lemma 9.2, for each $\mathbf {q}\in Q_{\varepsilon }$, there exists a unique solution $(\Phi _{\varepsilon ,\mathbf {q}},\Psi _{\varepsilon ,\mathbf {q}})\in \mathcal {K}_{\varepsilon ,\mathbf {q}}^{\perp }$ such that

$$\begin{aligned} S_{\varepsilon ,\mathbf {q}}\left( \begin{matrix}A_{\varepsilon ,\mathbf {q}}+\Phi _{\varepsilon ,\mathbf {q}}\\ H_{\varepsilon ,\mathbf {q}} +\Psi _{\varepsilon ,\mathbf {p}}\end{matrix}\right) =\left( \begin{matrix}\Xi _{\varepsilon ,\mathbf {q}}\\ 0\end{matrix}\right) \in \mathcal {C}_{\varepsilon ,\mathbf {q}}. \end{aligned}$$

Our idea is to find $\mathbf {q}$ such that $$S_{\varepsilon ,\mathbf {q}}\left( \begin{matrix}A_{\varepsilon ,\mathbf {q}}+\Phi _{\varepsilon ,\mathbf {q}}\\ H_{\varepsilon ,\mathbf {q}}+\Psi _{\varepsilon ,\mathbf {q}}\end{matrix}\right) \perp \mathcal {C}_{\varepsilon ,\mathbf {q}}$$. Let

$$\begin{aligned} W_{\varepsilon ,j,i}(\mathbf {q}):=\frac{1}{\xi }\int _{B_{R/\varepsilon }}\left( S_{1} \big (A_{\varepsilon ,\mathbf {q}}+\Phi _{\varepsilon ,\mathbf {q}},H_{\varepsilon ,\mathbf {q}}+\Psi _{\varepsilon ,\mathbf {q}}\big )\frac{\partial A_{\varepsilon ,\mathbf {q}}}{\partial q_{j,i}}\right) \,\mathrm {d}z, \end{aligned}$$

where $j=1,2,\ldots ,k$ and $i=1,2$. We set

$$\begin{aligned} W_{\varepsilon }(\mathbf {q})=\left( W_{\varepsilon ,1,1}(\mathbf {q}),\ldots ,W_{\varepsilon ,k,2}(\mathbf {q})\right) . \end{aligned}$$

It is easy to see that $W_{\varepsilon }(\mathbf {q})$ is a map which is continuous in $\mathbf {q}$, and our problem is reduced to finding a zero of the vector field $W_{\varepsilon }(\mathbf {q})$. Since the points $q_1,q_2,\ldots ,q_k$ are the vertices of a regular *k*-polygon and $\mu (\varepsilon |x|)$ is a radially symmetric function, if we can find $\mathbf {q}\in Q_{\varepsilon }$ such that $\left( W_{\varepsilon ,1,1}(\mathbf {q}),W_{\varepsilon ,1,2}(\mathbf {q})\right) =0$, then $W_{\varepsilon }(\mathbf {q})=0$. Further, we note that the approximate solution $(A_{\varepsilon ,\mathbf {q}},H_{\varepsilon ,\mathbf {q}})$ is invariant under rotation by $\frac{2\pi }{k}$. Recall that by (3.1), we have $q_1=(q_{1,1},0)=(2R_\varepsilon ,0)$. Thus, using [1, Corollary 7.1], $W_{\varepsilon ,1,2}$ equals 0. So, all that remains is finding $\mathbf {q}$ such that $W_{\varepsilon ,1,1}(\mathbf {q})=0$.

We calculate the asymptotic expansion of $W_{\varepsilon ,1,1}(\mathbf {q})$,

$$\begin{aligned}&\int _{B_{R/\varepsilon }}S_1(A_{\varepsilon ,\mathbf {q}}+\Phi _{\varepsilon ,\mathbf {q}},H_{\varepsilon ,\mathbf {q}} +\Psi _{\varepsilon ,\mathbf {q}})\frac{\partial A_{\varepsilon ,\mathbf {q}}}{\partial q_{1,1}}\,\mathrm {d}z\nonumber \\&\quad =\int _{B_{R/\varepsilon }}\left[ \Delta (A_{\varepsilon ,\mathbf {q}}+\Phi _{\varepsilon ,\mathbf {q}}) -\mu (A_{\varepsilon ,\mathbf {q}}+\Phi _{\varepsilon ,\mathbf {q}}) +\frac{(A_{\varepsilon ,\mathbf {q}}+\Phi _{\varepsilon ,\mathbf {q}})^2}{H_{\varepsilon ,\mathbf {q}}+\Psi _{\varepsilon ,\mathbf {q}}} \right] \frac{\partial A_{\varepsilon ,\mathbf {q}}}{\partial q_{1,1}}\,\mathrm {d}z\nonumber \\&\quad =\int _{B_{R/\varepsilon }}\left[ \Delta (A_{\varepsilon ,\mathbf {q}}+\Phi _{\varepsilon ,\mathbf {q}}) -(A_{\varepsilon ,\mathbf {q}}+\Phi _{\varepsilon ,\mathbf {q}}) +\frac{(A_{\varepsilon ,\mathbf {q}}+\Phi _{\varepsilon ,\mathbf {q}})^2}{H_{\varepsilon ,\mathbf {q}}}\right] \frac{\partial A_{\varepsilon ,\mathbf {q}}}{\partial q_{1,1}}\,\mathrm {d}z\nonumber \\&\qquad +\int _{B_{R/\varepsilon }}\left[ \frac{(A_{\varepsilon ,\mathbf {q}} +\Phi _{\varepsilon ,\mathbf {q}})^2}{H_{\varepsilon ,\mathbf {q}}+\Psi _{\varepsilon ,\mathbf {q}}}- \frac{(A_{\varepsilon ,\mathbf {q}}+\Phi _{\varepsilon ,\mathbf {q}})^2}{H_{\varepsilon ,\mathbf {q}}}\right] \frac{\partial A_{\varepsilon ,\mathbf {q}}}{\partial q_{1,1}}\,\mathrm {d}z \nonumber \\&\qquad +\int _{B_{R/\varepsilon }}\left[ (1-\mu )(A_{\varepsilon ,\mathbf {q}}+\Phi _{\varepsilon ,\mathbf {q}}) \right] \frac{\partial A_{\varepsilon ,\mathbf {q}}}{\partial q_{1,1}}\,\mathrm {d}z\nonumber \\&\quad =I_1+I_2+I_3, \end{aligned}$$

where $I_i,i=1,2,3$ are defined at the last equality.

For $I_1$, we have by Lemma 9.2,

4.1 $$\begin{aligned} I_1&=\int _{B_{R/\varepsilon }}\left[ \Delta (A_{\varepsilon ,\mathbf {q}}+\Phi _{\varepsilon ,\mathbf {q}}) -(A_{\varepsilon ,\mathbf {q}}+\Phi _{\varepsilon ,\mathbf {q}})+\frac{(A_{\varepsilon ,\mathbf {q}}+\Phi _{\varepsilon ,\mathbf {q}})^2}{H_{\varepsilon ,\mathbf {q}}(q_1)}\right] \frac{\partial A_{\varepsilon ,\mathbf {q}}}{\partial q_{1,1}}\,\mathrm {d}z\nonumber \\&-\int _{B_{R/\varepsilon }}\frac{(A_{\varepsilon ,\mathbf {q}}+\Phi _{\varepsilon ,\mathbf {q}})^2}{H_{\varepsilon ,\mathbf {q}}^2(q_1)}(H_{\varepsilon ,\mathbf {q}}-H_{\varepsilon ,\mathbf {q}}(q_1))\frac{{\partial A_{\varepsilon ,\mathbf {q}}}}{\partial q_{1,1}}\,\mathrm {d}z+O\left( e^{-2R_{\varepsilon }\sin \frac{\pi }{k}}\right) \nonumber \\&=-\int _{B_{R/\varepsilon }}\left[ \Delta (w_1+\Phi _{\varepsilon ,\mathbf {q}})-(w_1+\Phi _{\varepsilon ,\mathbf {q}}) +\frac{(w_1+\Phi _{\varepsilon ,\mathbf {q}})^2}{H_{\varepsilon ,\mathbf {q}}(q_1)}\right] \frac{\partial w_1}{\partial z_{1}}\,\mathrm {d}z\nonumber \\&+\int _{B_{R/\varepsilon }}\frac{(w_1+\Phi _{\varepsilon ,\mathbf {q}})^2}{H^2_{\varepsilon }(q_1)^2} (H_{\varepsilon ,\mathbf {q}}(q_1+z)-H_{\varepsilon ,\mathbf {q}}(q_1))\frac{\partial w_1}{\partial z_1}\,\mathrm {d}z+O\left( e^{-2R_{\varepsilon }\sin \frac{\pi }{k}}\right) , \end{aligned}$$

where $w_1(z)=w(q_1+z)$. Note that by Lemma 9.2, we have $\Phi _{\varepsilon ,\mathbf {q},2}$ is radially symmetric with respect to *z*. Then we have

4.2 $$\begin{aligned} \int _{B_{R/\varepsilon }}\Big [\Delta \Phi _{\varepsilon ,\mathbf {q}}-\Phi _{\varepsilon ,\mathbf {q}}+2w_{1}\Phi _{\varepsilon ,\mathbf {q}}\Big ]\frac{\partial w_{1}}{\partial z_1}\,\mathrm {d}z&=\int _{B_{R/\varepsilon }}\Phi _{\varepsilon ,\mathbf {q},1}\frac{\partial }{\partial z_1}[\Delta w-w+w^2]\,\mathrm {d}z=0 \end{aligned}$$

and

4.3 $$\begin{aligned} \int _{B_{R/\varepsilon }}(\Phi _{\varepsilon ,\mathbf {q}})^2\frac{\partial w_1}{\partial z_1}\,\mathrm {d}z&= \int _{B_{R/\varepsilon }}\Phi _{\varepsilon ,\mathbf {q},1} \Phi _{\varepsilon ,\mathbf {q},2}\frac{\partial w_1}{\partial z_1}\,\mathrm {d}z\nonumber \\&=O\left( \sigma \left( \log \frac{1}{\sigma }\right) ^{-2}R_{\sigma }^{-\frac{1}{2}}e^{-R_{\sigma }}+ \sigma ^2\left( \log \frac{1}{\sigma }\right) ^{-2}\right. \nonumber \\&\left. +\left( \log \frac{1}{\sigma }\right) ^{-1}\varepsilon ^2R_{\varepsilon }\right) . \end{aligned}$$

From (4.1)–(4.3), we get

4.4 $$\begin{aligned} I_1&=\int _{B_{R/\varepsilon }}w_1^2(H_{\varepsilon ,\mathbf {q}}(q_1+z)-H_{\varepsilon ,\mathbf {q}}(q_1)) \frac{\partial w_1}{\partial z_1}\,\mathrm {d}z+h.o.t.\nonumber \\&=\xi \sigma \sum _{k=1}^2\frac{\partial F(\mathbf {q})}{\partial q_{1,k}} \int _{\mathbb {R}^2}w^2z_k\frac{\partial w}{\partial z_1}\,\mathrm {d}z\int _{\mathbb {R}^2}w^2\,\mathrm {d}z +h.o.t.\nonumber \\&=-c_1\xi \sigma \frac{\partial F(\mathbf {q})}{\partial q_{1,1}}+h.o.t., \end{aligned}$$

where $F(\mathbf {q})$ is defined in (3.15), $c_1=\frac{1}{3}\int _{\mathbb {R}^2}w^2\,\mathrm {d}z\int _{\mathbb {R}^2}w^3\,\mathrm {d}z$ and *h*.*o*.*t*. represent terms of the order

$$\begin{aligned} \sigma \left( \log \frac{1}{\sigma }\right) ^{-2}R_{\sigma }^{-\frac{1}{2}}e^{-R_{\sigma }} +\sigma ^2\left( \log \frac{1}{\sigma }\right) ^{-2} +\left( \log \frac{1}{\sigma }\right) ^{-1}\varepsilon ^2R_{\varepsilon }. \end{aligned}$$

Next we study the term $I_2$. We recall that $\Psi _{\varepsilon ,\mathbf {q}}$ satisfies the following equation

4.5 $$\begin{aligned} \Delta \Psi _{\varepsilon ,\mathbf {q}}-\sigma ^2\Psi _{\varepsilon ,\mathbf {q}}+2\xi A_{\varepsilon ,\mathbf {q}}\Phi _{\varepsilon ,\mathbf {q}}+\xi \Phi _{\varepsilon ,\mathbf {q}}^2=0. \end{aligned}$$

As for the perturbation term $\Phi _{\varepsilon ,\mathbf {q}}$, we can also make a decomposition for $\Psi _{\varepsilon ,\mathbf {q}}=\Psi _{\varepsilon ,\mathbf {q},1}+\Psi _{\varepsilon ,\mathbf {q},2}$, where

$$\begin{aligned} \Delta \Psi _{\varepsilon ,\mathbf {q},1}-\sigma ^2\Psi _{\varepsilon ,\mathbf {q},1}+2\xi A_{\varepsilon ,\mathbf {q}}\Phi _{\varepsilon ,\mathbf {q},1}+\xi \left( \Phi _{\varepsilon ,\mathbf {q},1}^2+2\Phi _{\varepsilon ,\mathbf {q},1} \Phi _{\varepsilon ,\mathbf {q},2}\right) =0, \end{aligned}$$

and

$$\begin{aligned} \Delta \Psi _{\varepsilon ,\mathbf {q},2}-\sigma ^2\Psi _{\varepsilon ,\mathbf {q},2}+2\xi A_{\varepsilon ,\mathbf {q}}\Phi _{\varepsilon ,\mathbf {q},2}+\xi \Phi _{\varepsilon ,\mathbf {q},2}^2=0. \end{aligned}$$

Then we can easily see that

$$\begin{aligned} \Vert \Psi _{\varepsilon ,\mathbf {q},1}\Vert _{H^2(B_{R/\varepsilon })}=O\left( \sigma \left( \log \frac{1}{\sigma }\right) ^{-1}R_{\sigma }^{-\frac{1}{2}}e^{-R_{\sigma }}+ \sigma ^2\left( \log \frac{1}{\sigma }\right) ^{-1}+\varepsilon ^2 R_{\varepsilon }\right) \end{aligned}$$

and $\Psi _{\varepsilon ,\mathbf {q},2}$ is radially symmetric with respect to *z*. Further, from the Green representation formula we get that

4.6 $$\begin{aligned} \Psi _{\varepsilon ,\mathbf {q},1}(q_1+z)-\Psi _{\varepsilon ,\mathbf {q}}(q_1)&=\xi \int _{B_{R/\varepsilon }} (G_{\sigma }(p_1,q_1+z)-G_\sigma (p_1,z)) \left( 2A_{\varepsilon ,\mathbf {q}}\Phi _{\varepsilon ,\mathbf {q}}+\Phi _{\varepsilon ,\mathbf {q}}^2\right) \hbox {d}z\nonumber \\&=o(1)\xi \sigma |\nabla q_1F(\mathbf {q})||z|+R_1(|z|), \end{aligned}$$

where $R_1(|z|)$ is a radially symmetric function.

Substituting (4.5) and (4.6) into $I_2$, we get

4.7 $$\begin{aligned} I_2&=\int _{B_{R/\varepsilon }}\left[ \frac{(A_{\varepsilon ,\mathbf {q}}+\Phi _{\varepsilon ,\mathbf {q}})^2}{H_{\varepsilon ,\mathbf {q}}+\Psi _{\varepsilon ,\mathbf {q}}}-\frac{(A_{\varepsilon }+\Phi _{\varepsilon ,\mathbf {q}})^2}{H_{\varepsilon ,\mathbf {q}}} \right] \frac{\partial A_{\varepsilon ,\mathbf {q}}}{\partial q_{1,1}}\,\mathrm {d}z\nonumber \\&=-\int _{B_{R/\varepsilon }}\frac{(A_{\varepsilon ,\mathbf {q}}+\Phi _{\varepsilon ,\mathbf {q}})^2}{H_{\varepsilon ,\mathbf {q}}^2}\Psi _{\varepsilon ,\mathbf {q}}\frac{\partial A_{\varepsilon ,\mathbf {q}}}{\partial q_{1,1}}\,\mathrm {d}z\nonumber \\&+O\left( \sigma \left( \log \frac{1}{\sigma }\right) ^{-2}R_{\sigma }^{-\frac{1}{2}}e^{-R_{\sigma }} +\sigma ^2\left( \log \frac{1}{\sigma }\right) ^{-2} +\left( \log \frac{1}{\sigma }\right) ^{-1}\varepsilon ^2R_{\varepsilon }\right) \nonumber \\&=-\int _{B_{R/\varepsilon }}\frac{1}{3}\frac{\partial w_{1}^3}{\partial y_1}(\Psi -\Psi (q_1))\,\mathrm {d}z\nonumber \\&+O\left( \sigma \left( \log \frac{1}{\sigma }\right) ^{-2}R_{\sigma }^{-\frac{1}{2}}e^{-R_{\sigma }} +\sigma ^2\left( \log \frac{1}{\sigma }\right) ^{-2} +\left( \log \frac{1}{\sigma }\right) ^{-1}\varepsilon ^2R_{\varepsilon }\right) \nonumber \\&=o(1)\xi \sigma |\nabla q_1F(\mathbf {q})|+O\left( \sigma \left( \log \frac{1}{\sigma }\right) ^{-2} R_{\sigma }^{-\frac{1}{2}}e^{-R_{\sigma }}+\sigma ^2\left( \log \frac{1}{\sigma }\right) ^{-2}\right. \nonumber \\&\left. +\left( \log \frac{1}{\sigma }\right) ^{-1}\varepsilon ^2R_{\varepsilon }\right) . \end{aligned}$$

For $I_3$, we have

4.8 $$\begin{aligned} I_3&=\int _{B_{R/\varepsilon }}(1-\mu )w(x-q_1)\frac{\partial w(x-q_1)}{\partial q_{1,1}}\,\mathrm {d}x+O\left( e^{-2R_{\varepsilon }\sin \left( \frac{\pi }{k}\right) }\right) \nonumber \\&=\int _{B_{R/\varepsilon }}(1-\mu (|\varepsilon q_1|))w_1\frac{\partial w_1}{\partial q_{1,1}}\,\mathrm {d}z+\int _{B_{R/\varepsilon }}(\mu (|\varepsilon q_1|) -\mu (|\varepsilon (q_1+z)|))w_1\frac{\partial w_1}{\partial q_{1,1}}\,\mathrm {d}z\nonumber \\&+O\left( e^{-2R_{\varepsilon }\sin \left( \frac{\pi }{k}\right) }\right) \nonumber \\&=\int _{\mathbb {R}^2}\Big [\frac{\partial \mu (|\varepsilon q_1|)}{\partial z_1} \varepsilon z_1+\frac{\partial \mu (|\varepsilon q_1|)}{\partial z_2}\varepsilon z_2 \Big ]w(z)\frac{\partial w(z)}{\partial z_1}\,\mathrm {d}z +O\left( e^{-2R_{\varepsilon }\sin \left( \frac{\pi }{k}\right) }\right) \nonumber \\&=\varepsilon \frac{\partial \mu (|\varepsilon q_1|)}{\partial z_1} \int _{\mathbb {R}^2}z_1 w\frac{\partial w}{\partial z_1}\,\mathrm {d}z +O\left( e^{-2R_{\varepsilon }\sin \left( \frac{\pi }{k}\right) }\right) \nonumber \\&=c_2\varepsilon ^2R_{\varepsilon }\mu ''(0)+O\left( e^{-2R_{\varepsilon }\sin \left( \frac{\pi }{k} \right) }+\varepsilon ^3 R_{\varepsilon }^2\right) , \end{aligned}$$

where $c_2=2\int _{\mathbb {R}^2}z_1 w\frac{\partial w}{\partial z_1}\,\mathrm {d}z<0$ is negative and $\mu ''(0)$ denotes the second radial derivative of the radially symmetric function $\mu$ at the origin.

From (4.1) to (4.8), we get that $W_{\varepsilon ,1,1}(\mathbf {q})$ can be represented as follows:

4.9 $$\begin{aligned} W_{\varepsilon ,1,1}(\mathbf {q})=-c_1\xi \sigma \frac{\partial F(\mathbf {q})}{\partial q_{1,1}}+c_2\varepsilon ^2R_{\varepsilon }\mu ''(0)+h.o.t. \end{aligned}$$

Using the asymptotic behaviour of the Green function $G_{\sigma }$, we have

4.10 $$\begin{aligned} W_{\varepsilon ,1,1}(\mathbf {q})&=-c_1\xi \sigma \partial _{q_{1,1}}(G_{\sigma }(q_1,q_2)+G_{\sigma }(q_1,q_k)) +c_2\varepsilon ^2R_{\varepsilon }\mu ''(0)+h.o.t.\nonumber \\&=c_1\xi \sigma R_{\sigma }^{-\frac{1}{2}}e^{-R_{\sigma }} \left( \frac{q_1-q_2}{|q_1-q_2|} +\frac{q_1-q_k}{|q_1-q_k|}\right) +c_2\varepsilon ^2R_{\varepsilon }\mu ''(0)+h.o.t.\nonumber \\&=2c_1\xi \sigma R_{\sigma }^{-\frac{1}{2}}e^{-R_{\sigma }}\sin \left( \frac{\pi }{k} \right) +c_2\varepsilon ^2R_{\varepsilon }\mu ''(0)+h.o.t. =:\widehat{W}_{\varepsilon ,1,1}(\mathbf {q})+h.o.t.. \end{aligned}$$

Thus, denoting the leading order contribution of $W_{\varepsilon ,1,1}(\mathbf {q})$ by $\widehat{W}_{\varepsilon ,1,1}(\mathbf {q})$, it follows that $\widehat{W}_{\varepsilon ,1,1}(\mathbf {q})$ depends only on $R_{\varepsilon }$. Then we have $\widehat{W}_{\varepsilon ,1,1}(\mathbf {q})=0$ for

4.11 $$\begin{aligned} \xi \left( 2\sigma R_{\varepsilon ,0}\sin \left( \frac{\pi }{k}\right) \right) ^{-\frac{3}{2}}e^{-2\sigma R_{\varepsilon ,0}\sin \left( \frac{\pi }{k}\right) }+c_3D=h.o.t., \end{aligned}$$

where $c_3=\frac{c_2\mu ''(0)}{4c_1(\sin \frac{\pi }{k})^2}$ is negative, and we finally get

$$\begin{aligned} R_{\varepsilon ,0}=\frac{1}{2\sigma \sin \left( \frac{\pi }{k}\right) } \left( \log \frac{1}{D} -\frac{3}{2}\log \log \frac{1}{D} -\log \frac{\xi }{c_3} +O\left( \frac{\log \log \frac{1}{D}}{\log \frac{1}{D}} \right) \right) . \end{aligned}$$

If $\xi ^{-1}D$ is sufficiently small, then we easily get that Eq. (4.11) admits a unique solution and it is nondegenerate. As a consequence, in the neighborhood of $R_{\varepsilon ,0}$, we can find ${\hat{R}}_{\varepsilon ,0}$ such that $W_{\varepsilon ,1,1}(\mathbf {q})=0$. Thus, we have solved the reduced problem and the proof of Theorem 1.1 is complete.

## Stability analysis I: study of large eigenvalues

To prove Theorem 1.2, we consider the stability of the solution $(A_{\varepsilon },H_{\varepsilon })$ for (1.2) which was given in Theorem 1.1.

Linearizing the Gierer–Meinhardt system (1.1) around the equilibrium states $(A_{\varepsilon },H_{\varepsilon })$, we obtain the following eigenvalue problem:

5.1 $$\begin{aligned} \left\{ \begin{array}{l} \Delta _x\phi _{\varepsilon }-\mu (|\varepsilon x|)\phi _{\varepsilon }+2\frac{A_{\varepsilon }}{H_{\varepsilon }}\phi _{\varepsilon }-\frac{A_{\varepsilon }^2}{H_{\varepsilon }^2}\psi _{\varepsilon }=\lambda _{\varepsilon }\phi _{\varepsilon },\\ \Delta _x\psi _{\varepsilon }-\sigma ^2\psi _{\varepsilon }+2\xi A_{\varepsilon }\phi _{\varepsilon }=\tau \lambda _{\varepsilon }\sigma ^2\psi _{\varepsilon }, \end{array}\right. \end{aligned}$$

Here $\lambda _{\varepsilon }$ is some complex number and

$$\begin{aligned} \phi _{\varepsilon }\in H^2_N(B_{R/\varepsilon }),\quad \psi _{\varepsilon }\in H^2_N(B_{R/\varepsilon }). \end{aligned}$$

In this section, we study the large eigenvalues, i.e., we assume that $|\lambda _{\varepsilon }|\ge c>0$ for $\varepsilon$ small. The derivation of a matrix characterizing the small eigenvalues will be done in appendix B since this study is quite technical. Finally, in the next section, we discuss the small eigenvalues explicitly by considering these matrices. That part is central to understanding the stability of spike clusters.

If $\mathfrak {R}(\lambda _{\varepsilon })\le -c$, we are done. Then $\lambda _{\varepsilon }$ is a stable large eigenvalue. Therefore we may assume that $\mathfrak {R}(\lambda _{\varepsilon })\ge -c$ and for a subsequence in $\varepsilon ,D$, we have $\lambda _{\varepsilon }\rightarrow \lambda _0\ne 0$. We shall derive the limiting eigenvalue problem of (5.1) as $\max \left( \frac{\varepsilon }{\sqrt{D}},D\log \frac{\sqrt{D}}{\varepsilon }\right) \rightarrow 0$ which reduces to a system of nonlocal eigenvalue problems.

The key references are Theorem 2.2 and Lemma 2.5.

The second equation in (5.1) is equivalent to

5.2 $$\begin{aligned} \Delta \psi _{\varepsilon }-\sigma ^2(1+\tau \lambda _{\varepsilon })\psi _{\varepsilon } +2\xi _{\varepsilon }A_{\varepsilon }\phi _{\varepsilon }=0. \end{aligned}$$

We introduce the following:

$$\begin{aligned} \sigma _{\lambda _{\varepsilon }}=\sigma \sqrt{1+\tau \lambda _{\varepsilon }}, \end{aligned}$$

where in $\sqrt{1+\tau \lambda _{\varepsilon }}$ we take the principal part of the square root. This means that the real part of $\sqrt{1+\tau \lambda _{\varepsilon }}$ is positive, which is possible because $\mathfrak {R}(1+\tau \lambda _{\varepsilon })\ge \frac{1}{2}$.

Let us assume that $\Vert \phi _{\varepsilon }\Vert _{H^2(B_{R/\varepsilon })}=1$. We cut off $\phi _{\varepsilon }$ as follows:

$$\begin{aligned} \phi _{\varepsilon ,j}(x)=\phi _{\varepsilon }(x)\chi _{\varepsilon ,q_j}(x), \end{aligned}$$

where the test function $\chi _{\varepsilon ,q_j}(x)$ was introduced in (3.3).

From $\mathfrak {R}(\lambda _{\varepsilon })\ge -c$ and the exponential decay of *w*, we can derive from (5.1) that

$$\begin{aligned} \phi _{\varepsilon }=\sum _{j=1}^k\phi _{\varepsilon ,j}+h.o.t.~\mathrm {in}~H^2_N(B_{R/\varepsilon }). \end{aligned}$$

Since $\Vert \phi _{\varepsilon }\Vert _{H^2(B_{R/\varepsilon })}=1$, by taking a subsequence, we may also assume that $\phi _{\varepsilon ,j}\rightarrow \phi _j$ in $H^2(B_{R/\varepsilon })$ as $\max \left( \frac{\varepsilon }{\sqrt{D}},D\log \frac{\sqrt{D}}{\varepsilon }\right) \rightarrow 0$ for $j=1,2,\ldots ,k$. We have by (5.2)

5.3 $$\begin{aligned} \psi _{\varepsilon }(x)=\xi \int _{B_{R/\varepsilon }}G_{\sigma _{\lambda _\varepsilon }}(x,z) A_{\varepsilon }(z)\phi _{\varepsilon }(z)\,\mathrm {d}z. \end{aligned}$$

At $x=q_i,~i=1,2,\ldots ,k$, we calculate

5.4 $$\begin{aligned} \psi _{\varepsilon }(q_i)=~&\xi \int _{B_{R/\varepsilon }}G_{\sigma _{\lambda _\varepsilon }} (q_i,z)\sum _{j=1}^k w_{j}(z)\phi _{\varepsilon ,j}(z)\,\mathrm {d}z+h.o.t.\nonumber \\ =~&\frac{1}{2\pi }\xi \log \frac{1}{\sigma _{\lambda _{\varepsilon }}} \int _{B_{R/\varepsilon }}w\phi \,\mathrm {d}z+h.o.t. \end{aligned}$$

Substituting the above equation in the first equation of (5.1), taking the limit $\max \left( \frac{\varepsilon }{\sqrt{D}},D\log \right. \left. \frac{\sqrt{D}}{\varepsilon }\right) \rightarrow 0$, we get

5.5 $$\begin{aligned} \Delta _x\phi _i-\phi _i+ 2w\phi _i- \frac{2}{1+\tau \lambda _0}\, \frac{\int _{\mathbb {R}^2} w\phi _i\,\mathrm {d}z}{\int _{\mathbb {R}^2} w^2\,\mathrm {d}z}w^2=\lambda _0\phi _i,~i=1,2\ldots ,k, \end{aligned}$$

where $\phi _i\in H^2(\mathbb {R}^2)$. Then we have

### Theorem 5.1

Let $\lambda _{\varepsilon }$ be an eigenvalue of (5.1) such that $\mathfrak {R}(\lambda _{\varepsilon })>-c$ for some $c>0$.

1. Suppose that (for suitable sequence $\max \left( \frac{\varepsilon }{\sqrt{D}},D\log \frac{\sqrt{D}}{\varepsilon }\right) \rightarrow 0$) we have $\lambda _{\varepsilon }\rightarrow \lambda _0\ne 0$. Then $\lambda _0$ is an eigenvalue of the nonlocal eigenvalue problem given in (5.5).
2. Let $\lambda _0\ne 0$ with $\mathfrak {R}(\lambda _0)>0$ be an eigenvalue of the nonlocal eigenvalue problem given in (5.5). Then for $\max \left( \frac{\varepsilon }{\sqrt{D}},D\log \frac{\sqrt{D}}{\varepsilon }\right)$ small enough, there is an eigenvalue $\lambda _{\varepsilon }$ of (5.1) with $\lambda _{\varepsilon }\rightarrow \lambda _0$ as $\max \left( \frac{\varepsilon }{\sqrt{D}},D\log \frac{\sqrt{D}}{\varepsilon }\right) \rightarrow 0$.

### Proof

(1) of Theorem 5.1 follows by asymptotic analysis similar to the one obtained in appendix A.

To prove (2) of Theorem 5.1, we follow a compactness argument of Dancer. For the details we refer to Chapter 4 of [26]. $\square$

We now study the stability of (5.1), by Lemma 2.5, for any nonzero eigenvalue $\lambda _0$ in (5.5) we have

$$\begin{aligned} \mathfrak {R}(\lambda _0)\le -c_0<0~\mathrm {for~some}~c_0>0. \end{aligned}$$

Thus, by Theorem 5.1, for $\max \left( \frac{\varepsilon }{\sqrt{D}},D\log \frac{\sqrt{D}}{\varepsilon }\right)$ small enough, all nonzero large eigenvalues of (5.1) have strictly negative real parts. More precisely, all eigenvalues $\lambda _{\varepsilon }$ of (5.1) for which $\lambda _{\varepsilon }\rightarrow \lambda _0\ne 0$ holds, satisfy $\mathfrak {R}(\lambda _{\varepsilon })\le -c<0$.

In conclusion, we have finished studying the large eigenvalues (of order *O*(1)) and derived results on their stability properties. It remains to study the small eigenvalues (of order *o*(1)) which will be done in appendix B and the next section.

## Stability analysis III: study of the matrix $M_{\mu }(\mathbf {q})$

In this section, we shall study the matrix $M_{\mu }(\mathbf {q})$ which was derived in appendix B. The eigenvalues of this matrix will determine the stability of small eigenvalues. Up to a constant positive factor, $M_{\mu }(\mathbf {q})$ is given by the hessian matrix of the term

6.1 $$\begin{aligned} {\prod ({\mathbf {q}})}= \sum _{i,j,\,i\ne j}\xi \frac{1}{(\sigma |q_i-q_j|)^{\frac{1}{2}}} e^{-\sigma |q_i-q_j|}+c_3\sum _{i=1}^k \mu (|\varepsilon q_i|), \end{aligned}$$

where $c_3=-\frac{c_2}{c_1}$ with $c_1,c_2$ given in (4.4) and (4.8) respectively.

Using

6.2 $$\begin{aligned} |q_1-q_2|=\frac{1}{\sigma } \left( \log \frac{1}{D} -\frac{3}{2}\log \log \frac{1}{D} -\log \frac{\xi }{c_3} +O\left( \frac{\log \log \frac{1}{D}}{\log \frac{1}{D}} \right) \right) , \end{aligned}$$

(see (4.11)), it is not difficult to see that in leading order

$$\begin{aligned} \frac{\partial ^2}{\partial q_{1,i}\partial q_{1,j}}{\prod ({\mathbf {q}})}&\sim \xi \sigma ^{\frac{3}{2}}e^{-\sigma |q_1-q_2|}\frac{1}{|q_1-q_2|^\frac{5}{2}}(q_1-q_2)_i(q_1-q_2)_j,\nonumber \\&+\xi \sigma ^{\frac{3}{2}}e^{-\sigma |q_1-q_k|}\frac{1}{|q_1-q_k|^\frac{5}{2}} (q_1-q_k)_i(q_1-q_k)_j, \end{aligned}$$

where $i,j=1,2$. By rotational symmetry, it is enough to compute

$$\begin{aligned} \frac{\partial ^2}{\partial q_{1,i}\partial q_{1,j}},\frac{\partial ^2}{\partial q_{1,i}\partial q_{2,j}},\frac{\partial ^2}{\partial q_{1,i}\partial q_{k,j}},~i,j=1,2. \end{aligned}$$

Terms which depend on $q_m$ are obtained by suitable rotation of terms which contain $q_1$. By straightforward computation, we have

$$\begin{aligned} \frac{\partial {\prod ({\mathbf {q}})}}{\partial q_{1,i}}&=-\xi \sigma ^{\frac{1}{2}}e^{-\sigma |q_1-q_2|}\frac{(q_1-q_2)_i}{|q_1-q_2|^{\frac{3}{2}}}- \xi \frac{1}{2\sigma ^{\frac{1}{2}}}e^{-\sigma |q_1-q_2|} \frac{(q_1-q_2)_i}{|q_1-q_2|^{\frac{5}{2}}}\\&-\xi \sigma ^{\frac{1}{2}}e^{-\sigma |q_1-q_k|}\frac{(q_1-q_k)_i}{|q_1-q_k|^{\frac{3}{2}}}-\xi \frac{1}{2\sigma ^{\frac{1}{2}}}e^{-\sigma |q_1-q_k|}\frac{(q_1-q_k)_i}{|q_1-q_k|^{\frac{5}{2}}}+h.o.t., \end{aligned}$$

and

6.3 $$\begin{aligned} \frac{\partial ^2{\prod ({\mathbf {q}})}}{\partial q_{1,i}\partial q_{1,j}}&=\xi \sigma ^{\frac{3}{2}}e^{-\sigma |q_1-q_2|}\frac{(q_1-q_2)_i(q_1-q_2)_j}{|q_1-q_2|^{\frac{5}{2}}}\nonumber \\&+\xi \sigma ^{\frac{3}{2}}e^{-\sigma |q_1-q_k|}\frac{(q_1-q_k)_i (q_1-q_k)_j}{|q_1-q_2|^{\frac{5}{2}}}+h.o.t.. \end{aligned}$$

For the terms $\frac{\partial ^2{\prod ({\mathbf {q}})}}{\partial q_{1,i}\partial q_{2,j}},~i,j=1,2$, we note that

$$\begin{aligned} \frac{\partial {\prod ({\mathbf {q}})}}{\partial q_{1,1}}=\xi \sigma ^{\frac{1}{2}}e^{-\sigma |q_2-q_1|}\frac{(q_2-q_1)_1}{|q_2-q_1|^{\frac{3}{2}}} +\xi \frac{1}{2\sigma ^{\frac{1}{2}}}e^{-\sigma |q_2-q_1|}\frac{(q_2-q_1)_1}{|q_2-q_1|^{\frac{5}{2}}}+h.o.t., \end{aligned}$$

and

$$\begin{aligned} \frac{\partial {\prod ({\mathbf {q}})}}{\partial q_{1,2}}=\xi \sigma ^{\frac{1}{2}} e^{-\sigma |q_2-q_1|}\frac{(q_2-q_1)_2}{|q_2-q_1|^{\frac{3}{2}}}+ \xi \frac{1}{2\sigma ^{\frac{1}{2}}}e^{-\sigma |q_2-q_1|}\frac{(q_2-q_1)_2}{|q_2-q_1|^{\frac{5}{2}}}+h.o.t.. \end{aligned}$$

Then we get

6.4 $$\begin{aligned} \frac{\partial ^2{\prod ({\mathbf {q}})}}{\partial q_{1,1}\partial q_{2,i}}=-\xi \sigma ^{\frac{3}{2}}e^{-\sigma |q_2-q_1|}\frac{(q_2-q_1)_1 (q_2-q_1)_i}{|q_2-q_1|^{\frac{5}{2}}}+h.o.t.,~i=1,2, \end{aligned}$$

and

6.5 $$\begin{aligned} \frac{\partial ^2{\prod ({\mathbf {q}})}}{\partial q_{1,2}\partial q_{2,i}}=-\xi \sigma ^{\frac{3}{2}}e^{-\sigma |q_1-q_2|}\frac{(q_2-q_1)_2 (q_2-q_1)_i}{|q_1-q_2|^{\frac{5}{2}}}+h.o.t.,~i=1,2. \end{aligned}$$

Similarly, for the terms $\frac{\partial ^2{\prod ({\mathbf {q}})}}{\partial q_{1,i}\partial q_{k,j}},~i,j=1,2$, we have

6.6 $$\begin{aligned} \frac{\partial ^2{\prod ({\mathbf {q}})}}{\partial q_{1,1}\partial q_{k,i}}=-\xi \sigma ^{\frac{3}{2}}e^{-\sigma |q_k-q_1|}\frac{(q_k-q_1)_1 (q_k-q_1)_i}{|q_k-q_1|^{\frac{5}{2}}}+h.o.t.,~i=1,2, \end{aligned}$$

and

6.7 $$\begin{aligned} \frac{\partial ^2{\prod ({\mathbf {q}})}}{\partial q_{1,2}\partial q_{k,i}}=-\xi \sigma ^{\frac{3}{2}}e^{-\sigma |q_k-q_1|}\frac{(q_k-q_1)_2 (q_k-q_1)_i}{|q_k-q_1|^{\frac{5}{2}}}+h.o.t.,~i=1,2. \end{aligned}$$

We now compute these expressions in a coordinate system of tangential and normal coordinates around each spike. We remark that these coordinates are the same as in [2]. The spike locations are given by

6.8 $$\begin{aligned} q_j^0= \left( \frac{R_\sigma }{ \sigma \sin \left( \frac{\pi }{k}\right) } \cos \theta _j, \frac{R_\sigma }{ \sigma \sin \left( \frac{\pi }{k}\right) } \sin \theta _j\right) ,\quad j=1,\ldots ,k, \end{aligned}$$

where

$$\begin{aligned} \theta _j=\frac{(j-1)2\pi }{k}+\alpha \end{aligned}$$

and $\alpha \in \mathbb {R}$. Note that the phase shift $\alpha$ appears in the problem due to the rotational invariance of $\mu =\mu (|y|)$ and we can choose $\alpha =0$. Then in local coordinates we can write

6.9 $$\begin{aligned} q_j=q_j^0+q_{j,1}\frac{q_j}{|q_j|}+q_{j,2}\frac{q_j^\perp }{|q_j^\perp |},\quad j=1,\ldots ,k, \end{aligned}$$

where $q_j$ is the radial (normal) vector and the tangential vector $q_j^\perp$ is obtained from $q_j$ by rotation of $\pi /2$ in anti-clockwise direction.

From (6.3) to (6.7), using the local coordinate frames and elementary trigonometry, the leading order of the matrix $M_{\mu }(\mathbf {q})$ is

6.10 $$\begin{aligned} M_{\mu }(\mathbf {q})&=\xi \sigma ^{\frac{3}{2}}e^{-\sigma |q_1-q_2|} \frac{1}{|q_1-q_2|^{\frac{5}{2}}} \left[ \begin{matrix} \left( \sin \frac{\pi }{k}\right) ^2(A_1+4I)&{}\sin \frac{\pi }{k}\cos \frac{\pi }{k}A_2\\ -\sin \frac{\pi }{k}\cos \frac{\pi }{k}A_2&{}-\left( \cos \frac{\pi }{k}\right) ^2A_1 \end{matrix}\right] +h.o.t., \end{aligned}$$

where

$$\begin{aligned} A_1=\left[ \begin{matrix} -2&{}\quad 1&{}\quad 0&{}\quad \cdots &{}\quad 0&{}\quad 1\\ 1&{}\quad -2&{}\quad 1&{}\quad \cdots &{}\quad 0&{}\quad 0\\ \vdots &{}\quad &{}\quad \vdots &{}\quad \vdots &{}\quad \ddots &{}\quad \vdots &{}\quad \vdots \\ 1&{}\quad 0&{}\quad 0&{}\quad \cdots &{}\quad 1&{}\quad -2 \end{matrix} \right] \quad \mathrm {and}\quad A_2=\left[ \begin{matrix} 0&{}\quad 1&{}\quad 0&{}\quad \cdots &{}\quad 0&{}\quad -1\\ -1&{}\quad 0&{}\quad 1&{}\quad \cdots &{}\quad 0&{}\quad 0\\ \vdots &{}\quad \vdots &{}\quad \vdots &{}\quad \ddots &{}\quad \vdots &{}\quad \vdots \\ 1&{}\quad 0&{}\quad 0&{}\quad \cdots &{}\quad -1&{}\quad 0 \end{matrix} \right] . \end{aligned}$$

Before analyzing the matrix in (6.10), we need some basic facts about circulant matrices. We follow the presentation in [3, 13] and include this material here for completeness. Denote the *k*-dimensional complex vector space and the ring of $k\times k$ complex matrices by $\mathbb {C}^k$ and $\mathbb {M}_k$, respectively. Let $\mathbf{b}=(b_1,b_2,\ldots ,b_k)\in \mathbb {C}^k$, we define a shift operator $S:\mathbb {C}^k\rightarrow \mathbb {C}^k$ by

$$\begin{aligned} S(b_1,b_2,\ldots ,b_k)=(b_k,b_1,\ldots ,b_{k-1}). \end{aligned}$$

### Definition 6.1

The circulant matrix $B=\text{ circ }(\mathbf{b})$ associated to the vector

$$\begin{aligned} \mathbf{b}=(b_1,b_2,\ldots ,b_k)\in \mathbb {C}^k \end{aligned}$$

is the $k\times k$ matrix whose *n*th row is $S^{n-1}{} \mathbf{b}$:

$$\begin{aligned} B=\left( \begin{matrix} b_1&{}\quad b_2&{}\quad \cdots &{}\quad b_{k-1}&{}\quad b_k\\ b_k&{}\quad b_1&{}\quad \cdots &{}\quad b_{k-2}&{}\quad b_{k-1}\\ \vdots &{}\quad \vdots &{}\quad \ddots &{}\quad \vdots &{}\quad \vdots \\ b_3&{}\quad b_4&{}\quad \cdots &{}\quad b_1&{}\quad b_2\\ b_2&{}\quad b_3&{}\quad \cdots &{}\quad b_k&{}\quad b_1 \end{matrix}\right) . \end{aligned}$$

We denote by $\text{ circ }(k)\subset \mathbb {M}_k$ the set of all $k\times k$ complex circulant matrices.

With this notation, both $A_1$ and $A_2$ are $k\times k$ circulant matrices. In fact,

$$\begin{aligned} A_1=\text{ circ }\{(-2,1,0,\ldots ,0,1)\}~\mathrm {and}~A_2 =\text{ circ }\{(0,1,0,\ldots ,0,-1)\}. \end{aligned}$$

Let $\epsilon =e^{\frac{2\pi i}{k}}$ be a primitive *k*th root of unity, we define

$$\begin{aligned} X_l=\frac{1}{\sqrt{k}}\left( 1,\epsilon ^{l},\epsilon ^{2l}, \ldots ,\epsilon ^{(k-1)l}\right) ^T\in \mathbb {C}^k, ~\mathrm {for}~l=0,\ldots ,k-1, \end{aligned}$$

and

$$\begin{aligned} P_k=\left( \begin{matrix} 1&{}\quad 1&{}\quad \cdots &{}\quad 1&{}\quad 1\\ 1&{}\quad \epsilon &{}\quad \cdots &{}\quad \epsilon ^{k-2}&{}\quad \epsilon ^{k-1}\\ \vdots &{}\quad \vdots &{}\quad \ddots &{}\quad \vdots &{}\quad \vdots \\ 1&{}\quad \epsilon ^{k-2}&{}\quad \cdots &{}\quad \epsilon ^{(k-2)^2}&{}\quad \epsilon ^{(k-2)(k-1)}\\ 1&{}\quad \epsilon ^{k-1}&{}\quad \cdots &{}\quad \epsilon ^{(k-1)(k-2)}&{}\quad \epsilon ^{(k-1)^2} \end{matrix}\right) . \end{aligned}$$

For the circulant matrix $B=\text{ circ }(\mathbf{b})$, let

$$\begin{aligned} \lambda _l=b_1+b_2\epsilon ^l+\cdots +b_k\epsilon ^{(k-1)l}, ~\mathrm {for\,}{ l=0,\ldots ,k-1}. \end{aligned}$$

A simple computation shows that $BX_l=\lambda _lX_l$. Hence $\lambda _l$ is an eigenvalue of *B* with normalised eigenvector $X_l$. Since $\{X_1,\ldots ,X_k\}$ is a linearly independent set of vectors in $\mathbb {C}^k$, all of the eigenvalues of *B* are given by $\lambda _l,~l=0,\ldots ,k-1$. By direct computation, the eigenvalues of $A_1$ are

$$\begin{aligned} \lambda _{1,l}=-2+\epsilon ^l+\epsilon ^{(k-1)l}=-4\sin ^2 \frac{l\pi }{k},~\mathrm {for}~l=0,\ldots ,k-1, \end{aligned}$$

and the eigenvalues of $A_2$ are

$$\begin{aligned} \lambda _{2,l}=\epsilon ^l-\epsilon ^{(k-1)l}=2i\sin \frac{2l\pi }{k}, ~\mathrm {for}~l=0,\ldots ,k-1. \end{aligned}$$

Let $\text{ diag }(a_1,a_2,\ldots ,a_k)$ denote the diagonal matrix with diagonal entries $a_1,a_2,\ldots ,a_k$ and

$$\begin{aligned} \mathcal {M}=\left[ \begin{matrix} \left( \sin \frac{\pi }{k}\right) ^2(A_1+4I)&{}\sin \frac{\pi }{k}\cos \frac{\pi }{k}A_2\\ -\sin \frac{\pi }{k}\cos \frac{\pi }{k}A_2&{}-\left( \cos \frac{\pi }{k}\right) ^2A_1 \end{matrix}\right] . \end{aligned}$$

From the above discussion for the circulant matrix, using

$$\begin{aligned} P=\left[ \begin{matrix} P_k&{}\quad 0_k\\ 0_k&{}\quad P_k \end{matrix}\right] \end{aligned}$$

and

$$\begin{aligned} 0_k=\text{ diag }\left( 0,0,0,\ldots ,0\right) , \end{aligned}$$

we have

$$\begin{aligned} P^{-1}\mathcal {M}P&= \left[ \begin{matrix} P_k^{-1}&{}0_k\\ 0_k&{}P_k^{-1} \end{matrix}\right] \left[ \begin{matrix} \left( \sin \frac{\pi }{k}\right) ^2(A_1+4I)&{}\sin \frac{\pi }{k}\cos \frac{\pi }{k}A_2\\ -\sin \frac{\pi }{k}\cos \frac{\pi }{k}A_2&{}-\left( \cos \frac{\pi }{k}\right) ^2A_1 \end{matrix}\right] \left[ \begin{matrix} P_k&{}0_k\\ 0_k&{}P_k \end{matrix}\right] \nonumber \\&=\left[ \begin{matrix} 4\left( \sin \frac{\pi }{k}\right) ^2(I-D_1)&{}i\sin \frac{2\pi }{k}D_2\\ -i\sin \frac{2\pi }{k}D_2&{}4\left( \cos \frac{\pi }{k}\right) ^2D_1 \end{matrix} \right] , \end{aligned}$$

where

$$\begin{aligned} D_1=\text{ diag }\left( 0,\left( \sin \frac{\pi }{k}\right) ^2,\left( \sin \frac{2\pi }{k}\right) ^2, \ldots ,\left( \sin \frac{(k-1)\pi }{k}\right) ^2\right) , \end{aligned}$$

and

$$\begin{aligned} D_2=\text{ diag }\left( 0,\sin \frac{2\pi }{k},\sin \frac{4\pi }{k}, \ldots ,\sin \frac{2(k-1)\pi }{k}\right) . \end{aligned}$$

Next we divide the matrix $P^{-1}\mathcal {M}P$ into *k* two by two matrices, where the $l-$th matrix ($l=0,1,\ldots ,k-1$) is given by

6.11 $$\begin{aligned} \left[ \begin{matrix} 4\left( \sin \frac{\pi }{k}\right) ^2\left( \cos \frac{l\pi }{k}\right) ^2&{}i\sin \frac{2\pi }{k} \sin \frac{2l\pi }{k}\\ -i\sin \frac{2\pi }{k}\sin \frac{2l\pi }{k}&{}4\left( \cos \frac{\pi }{k}\right) ^2 \left( \sin \frac{l\pi }{k}\right) ^2 \end{matrix}\right] . \end{aligned}$$

It is easy to see that the determinant of the above matrix is 0 and its trace is positive. Further, we see that the zero eigenvector of the above matrix is

6.12 $$\begin{aligned} \left( \cos \frac{\pi }{k}\sin \frac{l\pi }{k}, i\sin \frac{\pi }{k}\cos \frac{l\pi }{k}\right) ^T. \end{aligned}$$

Since the leading order matrix $\mathcal {M}$ admits zero eigenvalues with geometric multiplicity *k*, we have to expand the matrix $M_{\mu }(\mathbf {q})$ to the next order to determine if these small eigenvalues have positive or negative real part.

Before doing that, we point out a useful fact. Let us consider for example the term $\frac{\partial ^2{\prod ({\mathbf {q}})}}{\partial q_{2,1}\partial q_{1,1}}$. By direct computation we get

6.13 $$\begin{aligned} \begin{aligned} \frac{\partial {\prod ({\mathbf {q}})}}{\partial q_{1,1}}&=\xi \sigma ^{\frac{1}{2}}e^{-\sigma |q_1-q_2|} \frac{1}{|q_2-q_1|^{\frac{3}{2}}}(q_2-q_1)_1\\&={\tilde{K}}(|q_1-q_2|)(q_2-q_1)_1. \end{aligned} \end{aligned}$$

Computing another derivative of ${\tilde{K}}(|q_1-q_2|)(q_2-q_1)_1$ with respect to $q_{2,1}$, we note that there are two types of terms:

$$\begin{aligned} \left[ \frac{\partial {\tilde{K}}(|q_1-q_2|)}{\partial q_{2,1}}\right] (q_2-q_1)_1(q_2-q_1)_1 \quad \text{ and } \quad {\tilde{K}}(|q_1-q_2|)\frac{\partial (q_2-q_1)_1}{\partial q_{2,1}}. \end{aligned}$$

The first term is of the same symmetry class as the leading order term (i.e., the higher order term differs from the leading order term only by some small factor). Therefore, this term can be absorbed into the leading-order matrix $\mathcal {M}$.

However, the second term is different and it has to be taken into account. In fact, we will see that this type of terms can be used to resolve the stability problem. We can re-write the second term as follows:

6.14 $$\begin{aligned} {\tilde{K}}(|q_1-q_2|)\frac{\partial (q_2-q_1)_1}{\partial q_{2,1}}=-{\tilde{K}}(|q_1-q_2|)\frac{1}{2}\frac{\partial ^2}{\partial q_{2,1}\partial q_{1,1}}|q_2-q_1|^2. \end{aligned}$$

Hence, up to some factors it is enough for us to consider the terms $\frac{1}{2}\frac{\partial ^2}{\partial q_{2,j}\partial q_{1,i}}|q_1-q_2|^2,~i,j=1,2$. These terms together with $c_3\varepsilon ^2\mu ''(0)$ are the next order terms in the matrix $M_{\mu }(\mathbf {q})$.

Using the local coordinate frames of $q_1$ and $q_2$ to express Cartesian local coordinates $x_{i,j},\,i,j,=1,2$, we get

$$\begin{aligned} x_{1,1}= & {} q_{1,1},\quad x_{1,2}=q_{1,2},\\ x_{2,1}= & {} q_{2,1}\cos \frac{2\pi }{k}-q_{2,2}\sin \frac{2\pi }{k},\quad x_{2,2}=q_{2,1}\sin \frac{2\pi }{k}+q_{2,2}\cos \frac{2\pi }{k}. \end{aligned}$$

Using (6.8) and (6.9), this implies

$$\begin{aligned} |q_1-q_2|^2&=\left| \left( q_1^0+q_{1,1}\frac{q_1}{|q_1|}+q_{1,2} \frac{q_1^\perp }{|q_1^\perp |}\right) -\left( q_2^0+q_{2,1}\frac{q_2}{|q_2|}+q_{2,2}\frac{q_2^\perp }{|q_2^\perp |}\right) \right| ^2\nonumber \\&=\left( R_{\sigma }\cos \frac{\pi }{k}+x_{2,2}-x_{1,2}\right) ^2 +\left( R_\sigma \sin \frac{\pi }{k}- x_{2,1}+x_{1,1}\right) ^2\nonumber \\&=R_{\sigma }^2+2R_{\sigma }\left( \cos \frac{2\pi }{k}q_{2,2} +\sin \frac{2\pi }{k}q_{2,1}-q_{1,2}\right) \cos \frac{\pi }{k}\nonumber \\&+2R_{\sigma }\left( -\cos \frac{2\pi }{k}q_{2,1}+\sin \frac{2\pi }{k}q_{2,2} +q_{1,1}\right) \sin \frac{\pi }{k}\nonumber \\&+\left( \cos \frac{2\pi }{k}q_{2,2}+\sin \frac{2\pi }{k}q_{2,1}-q_{1,2}\right) ^2\nonumber \\&+\left( -\cos \frac{2\pi }{k}q_{2,1}+\sin \frac{2\pi }{k}q_{2,2}+q_{1,1}\right) ^2\nonumber \\&=R_{\sigma }^2+2R_{\sigma }\left( \cos \frac{2\pi }{k}q_{2,2}+\sin \frac{2\pi }{k}q_{2,1}-q_{1,2}\right) \cos \frac{\pi }{k}\nonumber \\&+2R_{\sigma }\left( -\cos \frac{2\pi }{k}q_{2,1}+\sin \frac{2\pi }{k} q_{2,2}+q_{1,1}\right) \sin \frac{\pi }{k}\nonumber \\&+q_{1,1}^2+q_{1,2}^2+q_{2,1}^2+q_{2,2}^2 -2q_{1,1}q_{2,1}\cos \frac{2\pi }{k} +2q_{1,1}q_{2,2}\sin \frac{2\pi }{k}\nonumber \\&-2q_{1,1}q_{2,2}\cos \frac{2\pi }{k} -2q_{1,2}q_{2,1}\sin \frac{2\pi }{k}. \end{aligned}$$

As a consequence, we have

6.15 $$\begin{aligned} \frac{\partial ^2|q_1-q_2|^2}{\partial ^2q_{i,j}}&=2,~i,j=1,2, \quad \frac{\partial ^2|q_1-q_2|^2}{\partial q_{1,1}\partial q_{2,1}} =\frac{\partial ^2|q_1-q_2|^2}{\partial q_{1,2}\partial q_{2,2}} =-2\cos \frac{2\pi }{k}, \end{aligned}$$

6.16 $$\begin{aligned} \frac{\partial ^2|q_1-q_2|^2}{\partial q_{1,1}\partial q_{2,2}}&=2\sin \frac{2\pi }{k},\, ~\frac{\partial ^2|q_1-q_2|^2}{\partial q_{1,2}\partial q_{2,1}}=-2\sin \frac{2\pi }{k},\, \frac{\partial ^2|q_1-q_2|^2}{\partial q_{1,1}\partial q_{1,2}}\nonumber \\&= ~\frac{\partial ^2|q_1-q_2|^2}{\partial q_{2,1}\partial q_{2,2}}=0. \end{aligned}$$

Similarly, in local coordinates $q_1,q_2$ we have

$$\begin{aligned} |q_1|^2=\left| q_1^0+q_{1,1}\frac{q_1}{|q_1|}+q_{1,2} \frac{q_1^\perp }{|q_1^\perp |}\right| ^2 =R_{\sigma }^2+q_{1,1}^2+q_{1,2}^2+2q_{1,1}|R_{\sigma }|, \end{aligned}$$

where we used $q_{1}\cdot q_1^\perp =0$. This implies

6.17 $$\begin{aligned} \frac{\partial ^2|q_1|^2}{\partial q_{1,1}^2}=\frac{\partial ^2|q_1|^2}{\partial q_{1,2}^2}=2,~ \frac{\partial ^2|q_1|^2}{\partial q_{1,1}\partial q_{1,2}}=0. \end{aligned}$$

For the terms $c_3\varepsilon ^2\mu ''(0)$, from (4.10) we derive that

6.18 $$\begin{aligned} 4\left( \sin \frac{\pi }{k}\right) ^2{\tilde{K}}(|q_1-q_2|)=c_3\varepsilon ^2\mu ''(0). \end{aligned}$$

From the above discussion and (6.13)–(6.18), expanding the matrix $M_{\mu }(\mathbf {q})$ we get the following second order contribution:

6.19 $$\begin{aligned} -{\tilde{K}}(|q_1-q_2|)\mathcal {M}_2 =-{\tilde{K}}(|q_1-q_2|) \left[ \begin{matrix} \cos \frac{2\pi }{k}A_1&{}\quad -\sin \frac{2\pi }{k}A_2\\ \sin \frac{2\pi }{k}A_2&{}\quad \cos \frac{2\pi }{k}A_1 \end{matrix}\right] . \end{aligned}$$

By using the matrix $P_k$, we diagonalise the matrix $\mathcal {M}_2$,

$$\begin{aligned} \left[ \begin{matrix} P_k^{-1}&{}\quad 0\\ 0&{}\quad P_k^{-1} \end{matrix}\right] \mathcal {M}_2 \left[ \begin{matrix} P_k&{}\quad 0\\ 0&{}\quad P_k \end{matrix}\right] =-{\tilde{K}}(|q_1-q_2|) \left[ \begin{matrix} -4\cos \frac{2\pi }{k}D_1&{}\quad -2i\sin \frac{2\pi }{k}D_2\\ 2i\sin \frac{2\pi }{k}D_2 &{}\quad -4\cos \frac{2\pi }{k}D_1 \end{matrix}\right] . \end{aligned}$$

From the discussion of the leading-order matrix $P^{-1}\mathcal {M}P$, we know that the vectors

$$\begin{aligned} v_{l,1}=\left( 0,\ldots ,\underbrace{\cos \frac{\pi }{k}\sin \frac{l\pi }{k}}_{l+1},0,\ldots , \underbrace{i\sin \frac{\pi }{k}\cos \frac{l\pi }{k}}_{k+l+1},\ldots ,0\right) ^T,\quad (l=0,1,\ldots ,k-1) \end{aligned}$$

are the eigenvectors with zero eigenvalues of the diagonal form. To show the stability of the eigenvalues in the linear subspace spanned by these eigenvectors, we have to evaluate the bilinear form with respect to these eigenvectors and show that

$$\begin{aligned} \mu _l= \frac{\langle (P^{-1}\mathcal {M}_2P)v_{l,1},v_{l,1}\rangle }{\langle v_{l,1},v_{l,1}\rangle } \ge 0, \quad (l=0,1,\ldots ,k-1). \end{aligned}$$

If $\langle (P^{-1}\mathcal {M}_2P)v_{l,1},v_{l,1}\rangle = 0$ some further study is needed. We compute

$$\begin{aligned} \mu _l=\frac{\langle (P^{-1}\mathcal {M}_2P)v_{l,1},v_{l,1}\rangle }{\langle v_{l,1},v_{l,1}\rangle }&=-4\cos \frac{2\pi }{k}\left( \sin \frac{l\pi }{k}\right) ^2\\&+ \frac{4\sin \frac{2\pi }{k}\sin \frac{2l\pi }{k} \cos \frac{\pi }{k}\sin \frac{\pi }{k}\cos \frac{l\pi }{k}\sin \frac{l\pi }{k}}{ \left( \cos \frac{\pi }{k}\right) ^2\left( \sin \frac{l\pi }{k}\right) ^2 + \left( \sin \frac{\pi }{k}\right) ^2\left( \cos \frac{l\pi }{k}\right) ^2 }. \end{aligned}$$

Next we discuss when all eigenvalues are positive (linearly stable solution) or some eigenvalues are negative (linearly unstable solution).

For $l=0$ we have $\mu _l=0$. This eigenvalue and its eigenvector are connected to rotational invariance of solutions.

For $l=1$ we compute the numerator in the expression for $\mu _1$ as

$$\begin{aligned}&-8\cos \frac{2\pi }{k}\left( \sin \frac{\pi }{k}\right) ^4 \left( \cos \frac{\pi }{k}\right) ^2 +16 \left( \sin \frac{\pi }{k}\right) ^4\left( \cos \frac{\pi }{k}\right) ^4\\&\quad =8\left( \sin \frac{\pi }{k}\right) ^4\left( \cos \frac{\pi }{k}\right) ^2>0. \end{aligned}$$

For $l=k-1$ we compare with the case $l=1$. The terms $\sin \frac{2l\pi }{k}$ and $\cos \frac{l\pi }{k}$ change sign, the other terms are the same as for $l=1$. The result is the same as for $l=1$. The eigenvalues $l=1$ and $l=k-1$ together with their eigenvectors correspond to translations and they are stable.

For $l=2$ we compute the numerator of $\mu _2$ as

$$\begin{aligned}&-4\cos \frac{2\pi }{k}\left( \sin \frac{2\pi }{k}\right) ^2 \left[ \left( \cos \frac{\pi }{k}\right) ^2\left( \sin \frac{2\pi }{k}\right) ^2 + \left( \sin \frac{\pi }{k}\right) ^2\left( \cos \frac{2\pi }{k}\right) ^2\right] \\&\quad +4\sin \frac{2\pi }{k}\sin \frac{4\pi }{k} \cos \frac{\pi }{k}\sin \frac{\pi }{k}\cos \frac{2\pi }{k}\sin \frac{2\pi }{k}\\&\quad = -4\cos \frac{2\pi }{k}\left( \sin \frac{2\pi }{k}\right) ^2 \left[ \left( \cos \frac{\pi }{k}\right) ^2\left( \sin \frac{2\pi }{k}\right) ^2 + \left( \sin \frac{\pi }{k}\right) ^2\left( \cos \frac{2\pi }{k}\right) ^2\right. \\&\qquad \left. -\left( \sin \frac{2\pi }{k}\right) ^2 \left( \left( \cos \frac{\pi }{k}\right) ^2-\left( \sin \frac{\pi }{k}\right) ^2\right) \right] \\&\quad =-4\cos \frac{2\pi }{k}\left( \sin \frac{2\pi }{k}\right) ^2\left( \sin \frac{\pi }{k}\right) ^2. \end{aligned}$$

Thus $\mu _2>0$ for $k=3$, $\mu _2=0$ for $k=4$ and $\mu _2<0$ for $k=5,\,6,\ldots$.

The eigenvalue for $l=2$ and $k=4$ is zero in the first two leading orders. To decide if it possibly contributes to an instability, further expansions are required. This computation is beyond the scope of this paper. We expect that the eigenvalue will be stable and the cluster with 4 spikes on a regular polygon is linearly stable.

The eigenvalue for $l=2$ and $k=5,6,\ldots$ is negative and so the cluster with 5 or more spikes is linearly unstable.

In summary we have considered the small eigenvalues and shown the following: The clusters with 2 spikes or 3 spikes on a polygon are both linearly stable. The clusters with 5 or more spikes are linearly unstable. The borderline case is the cluster with 4 spikes for which one eigenvalue requires further investigation to determine its stability.

Next we consider clusters with spikes located on a regular polygon plus a spike in its centre.

## Cluster of spikes on a polygon with centre

In this section, we sketch how the approach can be adapted to show existence and stability of a cluster for which the spikes are located at the vertices of a regular *k*-polygon with centre. The spike positions are

7.1 $$\begin{aligned} Q_{\varepsilon }=\left\{ \mathbf{q}=(q_1,\ldots ,q_k,0)\mid q_i =\left( 2{\tilde{R}}_{\varepsilon }\cos \frac{2(i-1)\pi }{k}, 2{\tilde{R}}_{\varepsilon }\sin \frac{2(i-1)\pi }{k}\right) \right\} , \end{aligned}$$

where ${\tilde{R}}_{\varepsilon }$ is chosen such that

7.2 $$\begin{aligned} \frac{1}{C}\frac{\sqrt{D}}{\varepsilon }\log \left( \frac{1}{D\log \frac{\sqrt{D}}{\varepsilon }}\right) \le {\tilde{R}}_{\varepsilon }\le C\frac{\sqrt{D}}{\varepsilon }\log \left( \frac{1}{D\log \frac{\sqrt{D}}{\varepsilon }}\right) \end{aligned}$$

for some constant $C>1$ independent of $\varepsilon$ and *D*.

To get the radius for the equilibrium position, we compute

$$\begin{aligned} \tilde{W}_{\varepsilon ,1,1}(\mathbf{q})=c_1\xi \sigma {\tilde{R}}_\sigma ^{-1/2}e^{-{\tilde{R}}_\sigma } +c_2 \varepsilon ^2 {\tilde{R}}_\varepsilon \mu ''(0) +\text{ h.o.t. }=0, \end{aligned}$$

where ${\tilde{R}}_\sigma =\sigma {\tilde{R}}_\varepsilon$. We get

$$\begin{aligned} {\tilde{R}}_{\varepsilon ,0}=\frac{1}{\sigma }\left( \log \frac{1}{D} -\frac{3}{2}\log \log \frac{1}{D} -\log \frac{\xi }{c_3} +O\left( \frac{\log \log \frac{1}{D}}{\log \frac{1}{D}} \right) \right) . \end{aligned}$$

Due to symmetry we also have $\tilde{W}_{\varepsilon ,1,2}(\mathbf{q})=0$ and $\tilde{W}_{\varepsilon ,k+1,1}(\mathbf{q})= \tilde{W}_{\varepsilon ,k+1,2}(\mathbf{q})= 0$. From this we get the existence of a steady state of spikes located at the *k* vertices of a polygon and its centre, where *k* can be any natural number.

Next we consider the stability of this spike cluster steady state. We assume that $k\le 5$. We take the same rotated coordinates as above around the vertices of the polygon. For the origin located in the centre of the polygon we keep Cartesian coordinates $x_1$ and $x_2$.

The matrix $\mathcal {\tilde{M}}_\mu (\mathbf {q})$ is now given as follows:

$$\begin{aligned} {\mathcal {\tilde{M}}}_\mu (\mathbf {q})= & {} \xi \sigma ^{\frac{3}{2}}e^{-\sigma |q_1|}\frac{1}{|q_1|^{\frac{5}{2}}} \left[ \begin{matrix} {\tilde{M}}_1 &{} {\tilde{M}}_2\\ {\tilde{M}}_3 &{} {\tilde{M}}_4 \end{matrix}\right] + h.o.t. \\= & {} \xi \sigma ^{\frac{3}{2}}e^{-\sigma |q_1|}\frac{1}{|q_1|^{\frac{5}{2}}} {\mathcal {\tilde{M}}}+ h.o.t. , \end{aligned}$$

where

$$\begin{aligned} \tilde{M}_1&=\left[ \begin{matrix} 1&{}\quad 0&{}\quad 0&{}\quad \cdots &{}\quad 0&{}\quad -1\\ 0&{}\quad 1&{}\quad 0&{}\quad \cdots &{}\quad 0&{}\quad -\cos \frac{2\pi }{k}\\ 0&{}\quad 0&{}\quad 1&{}\quad \cdots &{}\quad 0&{}\quad -\cos \frac{4\pi }{k}\\ \vdots &{}\quad \vdots &{}\quad \vdots &{}\quad \ddots &{}\quad \vdots &{}\quad \vdots \\ -1 &{}\quad -\cos \frac{2\pi }{k}&{}\quad -\cos \frac{4\pi }{k}&{}\quad \cdots &{}\quad -\cos \frac{2(k-1)\pi }{k}&{}\quad \frac{k}{2} \end{matrix}\right] ,\\ \tilde{M}_2&=\left[ \begin{matrix} 0&{}\quad 0&{}\quad 0&{}\quad \cdots &{}\quad 0&{}\quad 0\\ 0&{}\quad 0&{}\quad 0&{}\quad \cdots &{}\quad 0&{}\quad -\sin \frac{2\pi }{k}\\ 0&{}\quad 0&{}\quad 0&{}\quad \cdots &{}\quad 0&{}\quad -\sin \frac{4\pi }{k}\\ \vdots &{}\quad \vdots &{}\quad \vdots &{}\quad \ddots &{}\quad \vdots &{}\quad \vdots \\ 0&{}\quad 0&{}\quad 0&{}\quad \cdots &{}\quad 0&{}\quad 0 \end{matrix} \right] ,\\ \tilde{M}_3&=\left[ \begin{matrix} 0&{}\quad 0&{}\quad 0&{}\quad \cdots &{}\quad 0&{}\quad 0\\ 0&{}\quad 0&{}\quad 0&{}\quad \cdots &{}\quad 0&{}\quad 0\\ 0&{}\quad 0&{}\quad 0&{}\quad \cdots &{}\quad 0&{}\quad 0\\ \vdots &{}\quad \vdots &{}\quad \vdots &{}\quad \ddots &{}\quad \vdots &{}\quad \vdots \\ 0&{}\quad -\sin \frac{2\pi }{k}&{}\quad -\sin \frac{4\pi }{k} &{}\quad \cdots &{}\quad -\sin \frac{2(k-1)\pi }{k}&{}\quad 0 \end{matrix}\right] ,\\ \tilde{M}_4&=\left[ \begin{matrix} 0&{}\quad 0&{}\quad 0&{}\quad \cdots &{}\quad 0&{}\quad 0\\ 0&{}\quad 0&{}\quad 0&{}\quad \cdots &{}\quad 0&{}\quad 0\\ 0&{}\quad 0&{}\quad 0&{}\quad \cdots &{}\quad 0&{}\quad 0\\ \vdots &{}\quad \vdots &{}\quad \vdots &{}\quad \ddots &{}\quad \vdots &{}\quad \vdots \\ 0&{}\quad 0&{}\quad 0&{}\quad \cdots &{}\quad 0&{}\quad \frac{k}{2} \end{matrix}\right] . \end{aligned}$$

We multiply $\mathcal {\tilde{M}}$ from the right by the vector $\mathbf {a}=(a_1,a_2,\ldots ,a_k,a_{k+1},0,0,\ldots ,0,a_{2k+2})^T$, i.e. we assume that the components $k+2,\,k+3,\ldots , 2k+1$ are all zero, and we also multiply $\mathcal {\tilde{M}}$ from the left by the transpose of this vector. Further, we set $a_{k+1}=\alpha ,\,a_{2k+2}=\beta$, where $\alpha$ and $\beta$ are some real numbers. Then we get

$$\begin{aligned} \mathbf {a}^T \mathcal {\tilde{M}}\mathbf {a}= \sum _{l=1}^{k} \left( a_l-\alpha \cos \frac{2(l-1)\pi }{k}-\beta \sin \frac{2(l-1)\pi }{k}\right) ^2. \end{aligned}$$

This means the matrix $\mathcal {\tilde{M}}$ is positive semi-definite if it is restricted to the components $1,2,\ldots ,k$, $k+1,2k+2$. The eigenvalue of any eigenvector in this class is always nonnegative. It is zero if and only if

$$\begin{aligned} a_l=\alpha \cos \frac{2(l-1)\pi }{k}+\beta \sin \frac{2(l-1)\pi }{k} \quad \text{ for } l=1,2,\ldots ,k, \end{aligned}$$

where $\alpha$ and $\beta$ are some real numbers which are independent of *l*. These eigenvectors have positive eigenvalues for the second-order part of the matrix. Similar to the computation for a polygon without centre it can be shown that there are positive contributions to the eigenvalues coming from the components $k+1$ and $2k+2$ which are related to the spike at the centre. Note that these eigenvectors correspond to translations.

In addition we have to study the eigenvalue of any eigenvector orthogonal to this class, i.e. for which the components $1,2,\ldots ,k,k+1,2k+2$ are zero and the components $k+2, \ldots , 2k+1$ are arbitrary. The leading-order matrix $\mathcal {M}$ for the cluster of the polygon without centre defined in Sect. 6 is the second-order contribution here. It is positive semi-definite in this class (since $A_1$ is positive semi-definite). It is strictly positive definite except for the eigenvector $(0,0,\ldots ,0,1,1,\ldots ,1)^T$ which has zero eigenvalue (since $A_1$ has zero eigenvector $(1,1,\ldots ,1)^T$). Note that this eigenvector corresponds to rotations. Here the components $k+2,\ldots ,2k+1$ for the polygon with central spike become the components $k+1,\ldots , 2k$ of the vector for the polygon without centre since the components $k+1$ and $2k+2$ are dropped.

These computations show that the eigenvalues of $\mathcal {\tilde{M}}$ are nonnegative and they are zero only for eigenvectors which correspond to the rotational invariance of the problem. Together we get the stability of the cluster with spikes located at the vertices of a regular polygon with $k\le 5$ vertices plus one spike at its centre.

## Discussion

We have shown the existence of spike clusters located near a nondegenerate minimum point of the precursor gradient for the Gierer–Meinhardt system such that the spikes are located on regular polygons. We have proved that these solutions are stable for two or three spikes and unstable for five or more spikes. We have considered the problem in the rotationally symmetric case. We have assumed that the precursor and the domain are both rotationally symmetric.

It will be interesting to extend these results to the case that the precursor and the domain are not rotationally symmetric. We are currently studying these effects using the approach in [2], where the existence of spiky patterns for the Schrödinger equation has been extended from the case of a rotationally symmetric potential to the general case. If $\mu$ is not rotationally symmetric generically there will be certain possible orientations of the spike cluster and we expect to have stable and unstable equilibrium orientations. If the domain is not a disk higher order terms coming from the regular part of the Green’s function will determine the orientation of the spike cluster and we expect to have stable and unstable equilibrium orientations. Because of the smallness of the inhibitor diffusivity we expect that the influence of $\mu$ will dominate that of the domain boundary.

Further analysis is needed to resolve the stability issue for a 4-spike cluster (regular polygon with 4 vertices). Further calculations are required to show that the $(k+1)$-spike cluster for $k\ge 6$ (regular polygon with *k* vertices plus a spike in the centre) is stable or unstable. The stability problem in this case requires some new analysis since the interaction between spikes is of a different type from the one considered in this paper. These issues are currently under investigation.

Whereas in one spatial dimension the spikes in a cluster are aligned with equal distance in leading order (although they differ in higher order) [27], in two spatial dimensions a variety of different spike configurations are possible. In this paper we have considered regular polygons and polygons with a spike in the centre. Other arrangements include concentric multiple polygons or positions close to regular polygons. Similar configurations have been studied in [1].

Biologically speaking, the precursor is the information retained from a previous stage of development and the patterns discovered in the reaction–diffusion system at the present will be able to determine the development in the future. The Gierer–Meinhardt system with precursor can be considered a minimal model to describe this behaviour. Generally one has to study larger systems which take into account other effects to make more reliable biological predictions. Therefore it will be interesting to consider reaction–diffusion systems of three and more components and investigate the role which spike clusters play in such systems. One such system is a consumer chain model for which existence and stability of a clustered spiky pattern has been investigated by the first two authors [25]. However, a more systematic approach will be needed to gain a better understanding of the role played by spike clusters in guiding biological development.

## Appendix A: Some results for Liapunov–Schmidt reduction in Sect. 3

In this appendix we will prove some results which are needed for Liapunov–Schmidt reduction in Sect. 3. In particular, we are going to show that the equation

$$\begin{aligned} \pi _{\varepsilon ,\mathbf {q}}\circ S_{\varepsilon ,\mathbf {q}}\left( \begin{matrix} A_{\varepsilon ,\mathbf {q}}+\phi _{\varepsilon ,\mathbf {q}}\\ H_{\varepsilon ,\mathbf {q}}+\psi _{\varepsilon ,\mathbf {q}} \end{matrix}\right) =0 \end{aligned}$$

has a unique solution $$\Sigma _{\varepsilon ,\mathbf {q}}=\left( \begin{matrix} \phi _{\varepsilon ,\mathbf {q}}\\ \psi _{\varepsilon ,\mathbf {q}} \end{matrix}\right) \in \mathcal {K}_{\varepsilon ,\mathbf {q}}^{\perp }$$ if $\max \left( \frac{\varepsilon }{\sqrt{D}},D\log \frac{\sqrt{D}}{\varepsilon }\right)$ is small enough.

Recall that

$$\begin{aligned} \mathcal {L}_{\varepsilon ,\mathbf {q}}=\pi _{\varepsilon ,\mathbf {q}}\circ {\tilde{L}}_{\varepsilon ,\mathbf {q}} :\mathcal {K}_{\varepsilon ,\mathbf {q}}^{\perp }\rightarrow \mathcal {C}_{\varepsilon ,\mathbf {q}}^{\perp }. \end{aligned}$$

We will first show that this linear operator is uniformly invertible. Then we will use this to prove the existence of $$\Sigma _{\varepsilon ,\mathbf {q}}=\left( \begin{matrix} \phi _{\varepsilon ,\mathbf {q}}\\ \psi _{\varepsilon ,\mathbf {q}} \end{matrix} \right) $$.

As a preparation, in the following two propositions we show the invertibility of the corresponding linearised operator $\mathcal {L}_{\varepsilon ,\mathbf {q}}$.

### Proposition 9.1

Let $\mathcal {L}_{\varepsilon ,\mathbf {q}}$ be given in (3.23). There exists a positive constant ${\bar{\delta }}$ such that for all $\max \left( \frac{\varepsilon }{\sqrt{D}},D\log \frac{\sqrt{D}}{\varepsilon }\right) \in (0,{\bar{\delta }})$, we can find a positive constant *C* which is independent of $\varepsilon ,D$ such that

9.1 $$\begin{aligned} \Vert \mathcal {L}_{\varepsilon ,\mathbf {q}}\Sigma \Vert _{L^2(B_{R/\varepsilon })} \ge C\Vert \Sigma \Vert _{H^2(B_{R/\varepsilon })} \end{aligned}$$

for arbitrary ${\mathbf {q}}\in Q_{\varepsilon },\Sigma \in \mathcal {K}_{\varepsilon ,\mathbf {q}}^{\perp }$. Further, the map $\mathcal {L}_{\varepsilon ,\mathbf {q}}$ is surjective.

### Proof of Proposition 9.1

Suppose (9.1) is false. Then there exist sequences $\{\varepsilon _n\},\{D_n\}$, $\{\mathbf {q}^n\}$, $\{\phi ^n\},\{\psi ^n\}$ such that $\max \left( \frac{\varepsilon _n}{\sqrt{D_n}},D_n\log \frac{\sqrt{D_n}}{\varepsilon _n}\right) \rightarrow 0$, $\mathbf {q}^n\in Q_{\varepsilon }$, $\phi ^n=\phi _{\varepsilon _n,D_n,\mathbf {q}^n},n=1,2,\ldots$ and

9.2 $$\begin{aligned}&\Vert \mathcal {L}_{\varepsilon _n,\mathbf {q}^n}\Sigma _n\Vert _{L^2(B_{R/\varepsilon })}\rightarrow 0~\mathrm {as}~n\rightarrow \infty ,\end{aligned}$$

9.3 $$\begin{aligned}&\Vert \phi ^n\Vert _{H^2(B_{R/\varepsilon })}+\Vert \psi ^n\Vert _{H^2(B_{R/\varepsilon })} =1,~n=1,2,\ldots . \end{aligned}$$

On the other hand, we note $\psi ^n$ satisfies

$$\begin{aligned} \Delta \psi ^n-\sigma ^2\psi ^n+2A_{\varepsilon _n,\mathbf {q}^n}\phi ^n=0. \end{aligned}$$

It is easy to see from the above equation we get $\Vert \psi ^n\Vert _{H^2(B_{R/\varepsilon })}\le C\Vert \phi ^n\Vert _{H^2(B_{R/\varepsilon })}$. Then we can assume $\Vert \phi ^n\Vert _{H^2(B_{R/\varepsilon })}=1$. We define $\phi _{n,i},i=1,2,\ldots ,k$ and $\phi _{n,k+1}$ as follows:

9.4 $$\begin{aligned} \phi _{n,i}(x)=\phi ^n(x)\chi \left( \frac{x-q_i}{R_{\varepsilon }\sin \frac{\pi }{k}}\right) ~\mathrm {and}~ \phi _{n,k+1}(x)=\phi ^n(x)-\sum _{i=1}^k\phi _{n,i}(x), \end{aligned}$$

where $\chi (x)$ is defined in (3.3). Since for $i=1,2,\ldots ,k$ each sequence $\{\phi _{n,i}\},~n=1,2,\ldots$ is bounded in $H^2_N(\mathbb {R}^2)$ it has a weak limit in $H^2_N(\mathbb {R}^2)$, and therefore also a strong limit in $L^2(\mathbb {R}^2)$ and $L^\infty (\mathbb {R}^2)$. Call this limit $\phi _i$. Then $\Phi =(\phi _1,\ldots ,\phi _k)^T$ solves the system $L\Phi =0$, where *L* is given in (2.6). By Lemma 2.4, $\Phi \in Ker(L)=K_0\oplus \cdots \oplus K_0$. Since $\phi ^n\perp K_{\varepsilon _n,\mathbf {q}_n}$, by taking $n\rightarrow \infty$ we get $\Phi \in Ker(L)^{\perp }$. Therefore, $\Phi =0$.

By elliptic estimates we have $\Vert \phi _{n,i}\Vert _{H^2(B_{R/\varepsilon })}\rightarrow 0$ as $n\rightarrow \infty$ for $i=1,2,\ldots ,k$. Further, since $\phi _{n,k+1}\rightarrow \phi _{k+1}$ we get

$$\begin{aligned} \Delta \phi _{k+1}-\phi _{k+1}=0~\mathrm {in}~B_{R/\varepsilon }. \end{aligned}$$

Therefore we conclude $\phi _{k+1}=0$ and $\Vert \phi _{n,k+1}\Vert _{H^2(B_{R/\varepsilon })}\rightarrow 0$ as $n\rightarrow \infty$. This contradicts to $\Vert \phi ^n\Vert _{H^2(B_{R/\varepsilon })}=1$.

To complete the proof of Proposition 9.1 we just need to show that conjugate operator to $\mathcal {L}_{\varepsilon ,\mathbf {q}}$ (denoted by $\mathcal {L}_{\varepsilon ,\mathbf {q}}^*$) is injective from $\mathcal {K}_{\varepsilon ,\mathbf {q}}^{\perp }$ to $\mathcal {C}_{\varepsilon ,\mathbf {q}}^{\perp }$ and the proof for $\mathcal {L}_{\varepsilon ,\mathbf {q}}^*$ follows almost the same process as for $\mathcal {L}_{\varepsilon ,\mathbf {q}}$ and we omit it. Thus we finish the proof of Proposition 9.1. $\square$

Now we are in position to solve the equation

9.5 $$\begin{aligned} \pi _{\varepsilon ,\mathbf {q}}\circ S_{\varepsilon ,\mathbf {q}}\left( \begin{matrix} A_{\varepsilon ,\mathbf {q}}+\phi \\ H_{\varepsilon ,\mathbf {q}}+\psi \end{matrix}\right) =0. \end{aligned}$$

Since $\mathcal {L}_{\varepsilon ,\mathbf {q}}\mid _{\mathcal {K}_{\varepsilon ,\mathbf {q}}^{\perp }}$ is invertible (call the inverse $\mathcal {L}_{\varepsilon ,\mathbf {q}}^{-1}$), we can rewrite (9.5) as

9.6 $$\begin{aligned} \Sigma =-\left( \mathcal {L}_{\varepsilon ,\mathbf {q}}^{-1}\circ \pi _{\varepsilon ,\mathbf {q}}\right) \left( S_{\varepsilon ,\mathbf {q}}\left( \begin{matrix} A_{\varepsilon ,\mathbf {q}}\\ H_{\varepsilon ,\mathbf {q}}\end{matrix}\right) \right) -\left( \mathcal {L}_{\varepsilon ,\mathbf {q}}^{-1} \circ \pi _{\varepsilon ,\mathbf {q}})(N_{\varepsilon ,\mathbf {q}}(\Sigma )\right) \equiv M_{\varepsilon ,\mathbf {q}}(\Sigma ), \end{aligned}$$

where

$$\begin{aligned} \Sigma =\left( \begin{matrix}\phi \\ \psi \end{matrix}\right) ,~ N_{\varepsilon ,\mathbf {q}}(\Sigma )=S_{\varepsilon ,\mathbf {q}}\left( \begin{matrix}A_{\varepsilon ,\mathbf {q}}+\phi \\ H_{\varepsilon ,\mathbf {q}} +\psi \end{matrix}\right) -S_{\varepsilon ,\mathbf {q}}\left( \begin{matrix}A_{\varepsilon ,\mathbf {q}}\\ H_{\varepsilon ,\mathbf {q}}\end{matrix}\right) -S_{\varepsilon ,\mathbf {q}}'\left( \begin{matrix}A_{\varepsilon ,\mathbf {q}}\\ H_{\varepsilon ,\mathbf {q}}\end{matrix}\right) \left[ \begin{matrix}\phi \\ \psi \end{matrix}\right] , \end{aligned}$$

and the operator $M_{\varepsilon ,\mathbf {q}}$ is defined by (9.6) for $\Sigma \in H^2_N(B_{R/\varepsilon })\times H^2_N(B_{R/\varepsilon })$. We are going to show the operator $M_{\varepsilon ,\mathbf {q}}$ is a contraction map in

$$\begin{aligned} B_{\varepsilon ,D}=\left\{ \Sigma \in H^2_N(B_{R/\varepsilon })\times H^2_N(B_{R/\varepsilon })\mid \Vert \Sigma \Vert _{H^2(B_{R/\varepsilon })}<\eta \right\} \end{aligned}$$

provided $\max \left( \frac{\varepsilon }{\sqrt{D}},D\log \frac{\sqrt{D}}{\varepsilon }\right)$ is small enough. We have by Lemma 3.1 and Proposition 9.1 that

$$\begin{aligned} \Vert M_{\varepsilon ,\mathbf {q}}(\Sigma )\Vert _{H^2)N(B_{R/\varepsilon })}\le&~ C\Big (\Vert \pi _{\varepsilon ,\mathbf {q}}\circ N_{\varepsilon ,\mathbf {q}}(\Sigma )\Vert _{L^2(B_{R/\varepsilon })}+\left\| \pi _{\varepsilon ,\mathbf {q}}\circ S_{\varepsilon ,\mathbf {q}}\left( \begin{matrix}A_{\varepsilon ,\mathbf {q}}\\ H_{\varepsilon ,\mathbf {q}}\end{matrix} \right) \right\| _{L^2(B_{R/\varepsilon })} \Big )\\ \le&~ C(c(\eta )\eta +c_{\varepsilon ,D}), \end{aligned}$$

where $C>0$ is independent of $\eta$, $c(\eta )\rightarrow 0$ as $\eta \rightarrow 0$ and $c_{\varepsilon ,D}\rightarrow 0$ as

$$\begin{aligned} \max \left( \frac{\varepsilon }{\sqrt{D}},D\log \frac{\sqrt{D}}{\varepsilon }\right) \rightarrow 0. \end{aligned}$$

Similarly we can show

$$\begin{aligned} \left\| M_{\varepsilon ,\mathbf {q}}(\Sigma )-M_{\varepsilon ,\mathbf {q}}(\Sigma ')\right\| _{H^2 (B_{R/\varepsilon })}\le Cc(\eta )\Vert \Sigma -\Sigma '\Vert _{H^2(B_{R/\varepsilon })}. \end{aligned}$$

If we choose $\eta$ sufficient small, then $M_{\varepsilon ,\mathbf {q}}$ is a contraction map on $B_{\varepsilon ,D}$. The existence of the fixed point $\Sigma _{\varepsilon ,\mathbf {q}}$ together with an error estimate now follow from the contraction mapping principle. Moreover $\Sigma _{\varepsilon ,\mathbf {q}}$ is a solution of (9.6).

Thus we have proved the following lemma.

### Lemma 9.2

There exists ${\bar{\delta }}>0$ such that for

$$\begin{aligned} \max \left( \frac{\varepsilon }{\sqrt{D}},D\log \frac{\sqrt{D}}{\varepsilon }\right) \in (0,{\bar{\delta }}) \end{aligned}$$

and $\mathbf {q}\in Q_{\varepsilon }$, we can find a unique $(\Phi _{\varepsilon ,\mathbf {q}},\Psi _{\varepsilon ,\mathbf {q}})\in \mathcal {K}_{\varepsilon ,\mathbf {q}}^{\perp }$ satisfying

$$\begin{aligned} S_{\varepsilon ,\mathbf {q}}\left( \begin{matrix}A_{\varepsilon ,\mathbf {q}}+\Phi _{\varepsilon ,\mathbf {q}}\\ H_{\varepsilon ,\mathbf {q}}+\Psi _{\varepsilon ,\mathbf {q}}\end{matrix}\right) \in \mathcal {C}_{\varepsilon ,\mathbf {q}} \end{aligned}$$

and

9.7 $$\begin{aligned} \Vert (\Phi _{\varepsilon ,\mathbf {q}},\Psi _{\varepsilon ,\mathbf {q}})\Vert _{H^2(B_{R/\varepsilon })}\le C\left( \frac{1}{\log \frac{\sqrt{D}}{\varepsilon }}+\varepsilon ^2 R_{\varepsilon }^2\right) . \end{aligned}$$

In the following, we need more refined estimates on $\Phi _{\varepsilon ,\mathbf {q}}$. We recall that $S_1$ can be decomposed into the two parts $S_{1,1}$ and $S_{1,2}$, where $S_{1,1}$ is in leading order an odd function and $S_{1,2}$ is in leading order a radially symmetric function. Similarly, we can decompose $\Phi _{\varepsilon ,\mathbf {q}}$:

### Lemma 9.3

Let $\Phi _{\varepsilon ,\mathbf {q}}$ be defined in Lemma 9.2. Then for $x=q_i+z,~|\sigma z|<\delta$ and $\delta >0$ small enough, we have

9.8 $$\begin{aligned} \Phi _{\varepsilon ,\mathbf {q}}=\Phi _{\varepsilon ,\mathbf {q},1}+\Phi _{\varepsilon ,\mathbf {q},2}, \end{aligned}$$

where $\Phi _{\varepsilon ,\mathbf {q},2}$ is a radially symmetric function in *z* and

9.9 $$\begin{aligned} \Vert \Phi _{\varepsilon ,\mathbf {q},1}\Vert _{H^2(B_{R/\varepsilon })}=O\left( \varepsilon ^2 R_{\varepsilon }+\sigma \left( \log \frac{1}{\sigma }\right) ^{-1}R_{\sigma }^{-\frac{1}{2}}e^{-R_{\sigma }} +\sigma ^2\left( \log \frac{1}{\sigma }\right) ^{-1}\right) . \end{aligned}$$

### Proof

Let $S[u]:=S_1(u,T[u])$. We first solve

9.10 $$\begin{aligned} S\big [A_{\varepsilon ,\mathbf {q}}+\Phi _{\varepsilon ,\mathbf {q},2}\big ]-S\big [A_{\varepsilon ,\mathbf {q}}\big ] +\sum _{j=1}^kS_{1,2}(x-q_j)\in C_{\varepsilon ,\mathbf {q}}, \end{aligned}$$

for $\Phi _{\varepsilon ,\mathbf {q},2}\in K_{\varepsilon ,\mathbf {q}}^{\perp }$. Then we solve

9.11 $$\begin{aligned} S\big [A_{\varepsilon ,\mathbf {q}}+\Phi _{\varepsilon ,\mathbf {q},2}+\Phi _{\varepsilon ,\mathbf {q},1}\big ] -S\big [A_{\varepsilon ,\mathbf {q}}+\Phi _{\varepsilon ,\mathbf {q},2}\big ]+\sum _{j=1}^kS_{1,1}(x-q_j)\in C_{\varepsilon ,\mathbf {q}}, \end{aligned}$$

for $\Phi _{\varepsilon ,\mathbf {q},1}\in K^{\perp }_{\varepsilon ,\mathbf {q}}$. Using the same proof as in Lemma 9.2, both Eqs. (9.10) and (9.11) have unique solution for $\max \left( \frac{\sqrt{D}}{\varepsilon },D\log \frac{\sqrt{D}}{\varepsilon }\right)$ sufficiently small. By uniqueness, $\Phi _{\varepsilon ,\mathbf {q}}=\Phi _{\varepsilon ,\mathbf {q},1}+\Phi _{\varepsilon ,\mathbf {q},2}$. It is easy to see that

$$\begin{aligned} \Vert S_{1,1}\Vert _{L^2(B_{R/\varepsilon })}=O\left( \varepsilon ^2 R_{\varepsilon }+\sigma \left( \log \frac{1}{\sigma }\right) ^{-1}R_{\sigma }^{-\frac{1}{2}}e^{-R_{\sigma }} +\sigma ^2\left( \log \frac{1}{\sigma }\right) ^{-1}\right) \end{aligned}$$

and $S_{1,2}\in C_{\varepsilon ,\mathbf {q}}^{\perp }$. Then we can conclude that $\Phi _{\varepsilon ,\mathbf {q},1}$ and $\Phi _{\varepsilon ,\mathbf {q},2}$ have the required properties. $\square$

## Appendix B: Stability analysis II: study of small eigenvalues

In this section, we shall study the small eigenvalues for Eq. (5.1). Namely, we assume $\lambda _{\varepsilon }\rightarrow 0$ as $\max \left( \frac{\varepsilon }{\sqrt{D}},D\log \frac{\sqrt{D}}{\varepsilon }\right) \rightarrow 0$. We shall show that the small eigenvalues are related to $\mu ''(0)$ and the Green function.

Again let $(A_{\varepsilon },H_{\varepsilon })$ be the equilibrium state constructed for equation (1.2). Let

$$\begin{aligned} A_{\varepsilon ,j}=\chi _{\varepsilon ,q_j}(x)A_{\varepsilon }(x),~j=1,2,\ldots ,k, \end{aligned}$$

where $\chi _{\varepsilon ,q_j}$ is defined before (3.3). Then it is easy to see that

10.1 $$\begin{aligned} A_{\varepsilon }=\sum _{j=1}^kA_{\varepsilon ,j}+h.o.t.~\mathrm {in}~H^2_N(B_{R/\varepsilon }). \end{aligned}$$

In last section, we have derived the nonlocal eigenvalue (5.5). Let us now set $\lambda _0=0$ in (5.5), we have that

10.2 $$\begin{aligned} \Delta \phi _i-\phi _i+2w\phi _i-2\frac{\int _{\mathbb {R}^2}w\phi _i\,\mathrm {d}x}{\int _{\mathbb {R}^2}w^2\,\mathrm {d}x}w^2=0, \end{aligned}$$

which is equivalent to

$$\begin{aligned} L_0\left( \phi _i-2\frac{\int _{\mathbb {R}^2}w\phi _i\,\mathrm {d}x}{\int _{\mathbb {R}^2}w^2\,\mathrm {d}x}w\right) =0,~i=1,\ldots ,k, \end{aligned}$$

where $L_0$ is defined in (2.2). By Lemma 2.1, we have

10.3 $$\begin{aligned} \phi _i-2\frac{\int _{\mathbb {R}^2}w\phi _i\,\mathrm {d}x}{\int _{\mathbb {R}^2}w^2\,\mathrm {d}x}w\in \mathrm {span}\left\{ \frac{\partial w}{\partial x_j},~j=1,2\right\} ,~i=1,2,\ldots ,k. \end{aligned}$$

Multiplying (10.3) by *w* and integrating over $\mathbb {R}^2$ and summing up, we have $\int _{\mathbb {R}^2}w\phi _i\,\mathrm {d}x=0$, and hence

10.4 $$\begin{aligned} \phi _j\in K_0=\mathrm {span}\left\{ \frac{\partial w}{\partial x_j},~j=1,2\right\} ,~i=1,2,\ldots ,k. \end{aligned}$$

(10.4) suggests that, at least formally, we should have

10.5 $$\begin{aligned} \phi _{\varepsilon }\sim \sum _{j=1}^k\sum _{i=1}^2a^{\varepsilon }_{j,i}\frac{\partial w}{\partial x_i}(x-q_j), \end{aligned}$$

where $a_{j,i}^{\varepsilon }$ are some constant coefficients.

Next we find a good approximation of $\frac{\partial w}{\partial x_i}(x-q_j)$. Note that $A_{\varepsilon ,j}(x)\sim w(x-q_j)$ in $H^2(B_{R/\varepsilon })$, and $A_{\varepsilon ,j}$ satisfies

$$\begin{aligned} \Delta A_{\varepsilon ,j}-\mu (|\varepsilon x|)A_{\varepsilon ,j}+\frac{A_{\varepsilon ,j}}{H_{\varepsilon }^2}+h.o.t.=0. \end{aligned}$$

Then we find $\frac{\partial A_{\varepsilon ,j}}{\partial x_i}$ satisfies

10.6 $$\begin{aligned} \Delta \frac{\partial A_{\varepsilon ,j}}{\partial x_i}-\mu (|\varepsilon x|)\frac{\partial A_{\varepsilon ,j}}{\partial x_i}+2\frac{A_{\varepsilon ,j}}{H_{\varepsilon }}\frac{\partial A_{\varepsilon ,j}}{\partial x_i} -\frac{A_{\varepsilon ,j}^2}{H^2_{\varepsilon }}\frac{\partial H_{\varepsilon }}{\partial x_i}-\varepsilon \mu '(|\varepsilon x|)\frac{x_i}{r}A_{\varepsilon ,j}+h.o.t.=0, \end{aligned}$$

and we have $\frac{\partial A_{\varepsilon ,j}}{\partial x_i}=(1+o(1))\frac{\partial w}{\partial x_i}(x-q_j)$.

We now decompose

10.7 $$\begin{aligned} \phi _{\varepsilon }=\sum _{j=1}^k\sum _{i=1}^2a_{j,i}^{\varepsilon }\frac{\partial A_{\varepsilon ,j}}{\partial x_i}+\phi _{\varepsilon }^{\perp } \end{aligned}$$

with complex numbers $a_{j,i}^{\varepsilon }$, where

10.8 $$\begin{aligned} \phi _{\varepsilon }^{\perp }\perp \tilde{K}_{\varepsilon ,\mathbf {q}}:=\mathrm {span}\left\{ \frac{\partial A_{\varepsilon ,j}}{\partial x_i}\mid j=1,2,\ldots ,k,~i=1,2\right\} . \end{aligned}$$

Our idea is to show that this is a good choice because the error $\phi ^{\perp }_{\varepsilon }$ is small in a suitable norm and thus can be neglected.

For the inhibitor eigenfunction $\psi _{\varepsilon }$, we make the following decomposition according to $\phi _{\varepsilon }$

10.9 $$\begin{aligned} \psi _{\varepsilon }=\sum _{j=1}^k\sum _{i=1}^2a_{j,i}^{\varepsilon }\psi _{\varepsilon ,j,i} +\psi _{\varepsilon }^{\perp }, \end{aligned}$$

where $\psi _{\varepsilon ,j,i}$ is the unique solution of the following problem,

10.10 $$\begin{aligned} \Delta \psi _{\varepsilon ,j,i}-\sigma ^2(1+\tau \lambda _{\varepsilon })\psi _{\varepsilon ,j,i} +2\xi _{\varepsilon }A_{\varepsilon ,j}\frac{\partial A_{\varepsilon ,j}}{\partial x_i}=0~\mathrm {in}~B_{R/\varepsilon }, \end{aligned}$$

and

10.11 $$\begin{aligned} \Delta \psi _{\varepsilon }^{\perp }-\sigma ^2(1+\tau \lambda _{\varepsilon })\psi _{\varepsilon }^{\perp } +2\xi _{\varepsilon }A_{\varepsilon }\phi _{\varepsilon }^{\perp }=0 ~\mathrm {in}~B_{R/\varepsilon }. \end{aligned}$$

Suppose that $\Vert \phi _{\varepsilon }\Vert _{H^2(B_{R/\varepsilon })}=1$. We have $a_{j,i}^{\varepsilon }=\frac{\int _{B_{R/\varepsilon }}\phi _{\varepsilon }\frac{\partial A_{\varepsilon ,j}}{\partial x_i}\,\mathrm {d}x}{\int _{\mathbb {R}^2}(\frac{\partial w}{\partial x_1})^2\,\mathrm {d}x}$, therefore, we get $|a_{j,i}^{\varepsilon }|\le C$.

Substituting the decomposition of $\phi _{\varepsilon }$ and $\psi _{\varepsilon }$ into the first equation in (5.1), we have

10.12 $$\begin{aligned} \begin{aligned}&\sum _{j=1}^k\sum _{i=1}^2a_{j,i}^{\varepsilon }\frac{A_{\varepsilon ,j}^2}{H_{\varepsilon }^2} \Big (-\psi _{\varepsilon ,j,i}+\frac{\partial H_{\varepsilon }}{\partial x_i}\Big ) +\varepsilon \sum _{j=1}^k\sum _{i=1}^2a_{j,i}^{\varepsilon } \frac{\partial \mu (|\varepsilon x|)}{\partial y_i}A_{\varepsilon ,j}\\&\quad +\Delta \phi _{\varepsilon }^{\perp }-\mu (|\varepsilon x|)\phi _{\varepsilon }^{\perp }+2\frac{A_{\varepsilon }}{H_{\varepsilon }}\phi _{\varepsilon }^{\perp } -\frac{A_{\varepsilon }^2}{H_{\varepsilon }^2}\psi _{\varepsilon }^{\perp }-\lambda _{\varepsilon }\phi ^{\perp }_{\varepsilon } +h.o.t.\\&\quad =\lambda _{\varepsilon }\sum _{j=1}^k\sum _{i=1}^2a_{j,i}^{\varepsilon }\frac{\partial A_{\varepsilon ,j}}{\partial x_i}. \end{aligned} \end{aligned}$$

We set

10.13 $$\begin{aligned} \mathcal {I}_1={\sum _{j=1}^k\sum _{i=1}^2a_{j,i}^{\varepsilon } \frac{A_{\varepsilon ,j}^2}{H^2_{\varepsilon }} \left( -\psi _{\varepsilon ,j,i}+\frac{\partial H_{\varepsilon }}{\partial x_i}\right) } +\varepsilon \sum _{j=1}^k\sum _{i=1}^2a_{j,i}^{\varepsilon } \frac{\partial \mu (|\varepsilon x|)}{\partial y_i} A_{\varepsilon ,j}, \end{aligned}$$

and

10.14 $$\begin{aligned} \mathcal {I}_2=\Delta \phi _{\varepsilon }^{\perp }-\mu (|\varepsilon x|)\phi _{\varepsilon }^{\perp }+2\frac{A_{\varepsilon }}{H_{\varepsilon }}\phi _{\varepsilon }^{\perp } -\frac{A_{\varepsilon }^2}{H_{\varepsilon }^2}\psi _{\varepsilon }^{\perp }-\lambda _{\varepsilon }\phi _{\varepsilon }^{\perp }. \end{aligned}$$

Next we shall first derive the estimate for $\phi _{\varepsilon }^\perp$. Using (10.12), since $\phi _{\varepsilon }^\perp \perp {\tilde{K}}_{\varepsilon ,\mathbf {q}}$, then similar to the proof of Proposition 9.1, we have that

10.15 $$\begin{aligned} \Vert \phi ^\perp _{\varepsilon }\Vert _{H^2(B_{R/\varepsilon })}\le C\Vert \mathcal {I}_1\Vert _{L^2(B_{R/\varepsilon })}. \end{aligned}$$

So our aim is to estimate the term $\mathcal {I}_1$. We note that

$$\begin{aligned} \frac{\partial H_{\varepsilon }}{\partial x_i}&=\xi \int _{B_{R/\varepsilon }} \frac{\partial }{\partial x_i}G_{\sigma }(x,z)A^2_{\varepsilon }(z)\,\mathrm {d}z\\&=\xi \int _{B_{R/\varepsilon }}\frac{\partial }{\partial x_i} (K_{\sigma }(x,z)+H_{\sigma }(x,z))A^2_{\varepsilon }(z)\,\mathrm {d}z+h.o.t. \end{aligned}$$

and (for $$\tau =0$$)

$$\begin{aligned} \psi _{\varepsilon ,j,i}(x)&=2\xi \int _{B_{R/\varepsilon }}G_{\sigma } (x,z)A_{\varepsilon ,j}\frac{\partial A_{\varepsilon ,j}}{\partial z_i}\,\mathrm {d}z\nonumber \\&=\xi \int _{B_{R/\varepsilon }}(K_{\sigma }(x,z)+H_{\sigma }(x,z) +o(\sigma ^2))\frac{\partial }{\partial z_i}A_{\varepsilon ,j}^2\,\mathrm {d}z, \end{aligned}$$

where $K_{\sigma }(x,z)$ and $H_{\sigma }(x,z)$ refer to the singular and the regular part of the Green function $G_{\sigma }(x,z)$, respectively. For $\tau >0$ the last formula should use $G_{\sigma _{\lambda _{\varepsilon }}}$ instead of $G_{\sigma }$. Since $\lambda _\varepsilon \rightarrow 0$ it can be shown by comparing the two Green functions that the error from this term does not contribute to the small eigenvalues in leading order. We omit the details.

Then from the above two formulas, we have

$$\begin{aligned} \frac{\partial H_{\varepsilon }}{\partial x_i}-\psi _{\varepsilon ,j,i}(x)&=\xi \int _{B_{R/\varepsilon }}\frac{\partial }{\partial x_i}K_{\sigma }(x,z)A_{\varepsilon ,j}^2(z)-K_{\sigma }(x,z)\frac{\partial }{\partial z_i}A_{\varepsilon ,j}^2\,\mathrm {d}z\nonumber \\&+\xi \int _{B_{R/\varepsilon }}\frac{\partial }{\partial x_i}H_{\sigma }(x,z)A_{\varepsilon ,j}^2(z)-H_{\sigma }(x,z)\frac{\partial }{\partial z_i}A_{\varepsilon ,j}^2\,\mathrm {d}z\nonumber \\&+\xi \int _{B_{R/\varepsilon }}\frac{\partial }{\partial x_i}\sum _{l,l\ne j}G_{\sigma }(x,z)A_{\varepsilon ,l}^2\,\mathrm {d}z+h.o.t.. \end{aligned}$$

Using the fact that

10.16 $$\begin{aligned} \frac{\partial }{\partial x_i}K_{\sigma }(x,z)+\frac{\partial }{\partial z_i}K_{\sigma }(x,z)=h.o.t., ~\mathrm {for}~x\ne z, \end{aligned}$$

and integrating by parts, we get

10.17 $$\begin{aligned} \frac{\partial H_{\varepsilon }}{\partial x_i}-\psi _{\varepsilon ,j,i}(x)=\xi \left( \frac{\partial }{\partial x_i}F_j(x)+h.o.t.\right) \int _{\mathbb {R}^2}w^2\,\mathrm {d}z, \end{aligned}$$

where $F_j(x)=H_{\sigma }(x,q_j)+\sum _{l, l\ne j} G_{\sigma }(x,q_l)$. Then from (10.13), we get

$$\begin{aligned} \mathcal {I}_1=\sum _{j=1}^k\sum _{i=1}^2 a_{j,i}^{\varepsilon }\left( \xi \frac{\partial F_j(q_j+\varepsilon x)}{\partial x_i}\int _{\mathbb {R}^2}w^2\,\mathrm {d}z+\varepsilon \mu '(|\varepsilon q_j|)\frac{x_i}{r}w(x)+h.o.t.\right) , \end{aligned}$$

where $r=\sqrt{x_1^2+x_2^2}$. Note that

$$\begin{aligned} \frac{\partial F_j(q_j)}{\partial x_i}= \frac{1}{2}\,\frac{\partial F(\mathbf {q})}{\partial q_{j,i}}. \end{aligned}$$

From the proof of Theorem 1.1, we observe that

$$\begin{aligned} w^2(x)\xi \frac{\partial F_j(x)}{\partial x_i}\int _{B_{R/\varepsilon }}w^2\,\mathrm {d}z +\varepsilon \mu '(|\varepsilon q_j|)\frac{x_i}{r}w(x)=o(\varepsilon ^2R_{\varepsilon }). \end{aligned}$$

Hence, we have

10.18 $$\begin{aligned} \Vert \mathcal {I}_1\Vert _{L^2(B_{R/\varepsilon }}=o\left( \varepsilon ^2R_{\varepsilon }\sum _{j=1}^k \sum _{i=1}^2|a_{j,i}^{\varepsilon }|\right) \end{aligned}$$

and

10.19 $$\begin{aligned} \Vert \phi _{\varepsilon }^\perp \Vert _{H^2(B_{R/\varepsilon })}\le C\Vert \mathcal {I}_1\Vert _{L^(B_{R/\varepsilon })} =o\left( \varepsilon ^2R_{\varepsilon }\sum _{j=1}^k\sum _{i=1}^2|a_{j,i}^{\varepsilon }|\right) . \end{aligned}$$

Using the equation for $\psi _{\varepsilon }^\perp$ and (10.19), we obtain that

10.20 $$\begin{aligned} \Vert \psi _{\varepsilon }^\perp \Vert _{L^\infty (\Omega )}=o\left( \varepsilon ^2 R_{\varepsilon }\sum _{j=1}^k\sum _{i=1}^2|a_{j,i}^{\varepsilon }|\right) . \end{aligned}$$

We calculate

10.21 $$\begin{aligned} \int _{B_{R/\varepsilon }}\mathcal {I}_2\frac{\partial A_{\varepsilon ,l}}{\partial x_m}\,\mathrm {d}x&=\int _{B_{R/\varepsilon }}\left( \frac{A_{\varepsilon ,l}^2}{H_{\varepsilon }^2} \left( \frac{\partial H_{\varepsilon }}{\partial x_m}\phi _{\varepsilon }^\perp -\frac{\partial A_{\varepsilon ,l}}{\partial x_m}\psi _{\varepsilon }^{\perp }\right) \right) \,\mathrm {d}x -\lambda _{\varepsilon }\int _{B_{R/\varepsilon }}\phi _{\varepsilon }^\perp \frac{\partial A_{\varepsilon ,l}}{\partial x_m}\,\mathrm {d}x\nonumber \\&=\int _{B_{R/\varepsilon }}\frac{A_{\varepsilon ,l}^2}{H_{\varepsilon }^2}\left( \frac{\partial H_{\varepsilon }}{\partial x_m}(q_l+x)-\frac{\partial H_{\varepsilon }}{\partial x_m}(q_l)\right) \phi _{\varepsilon }^\perp \,\mathrm {d}x\nonumber \\&+\int _{B_{R/\varepsilon }}\frac{A_{\varepsilon ,l}^2}{H_{\varepsilon }^2}\left( \frac{\partial H_{\varepsilon }}{\partial x_m}(q_l)\right) \phi _{\varepsilon }^\perp \,\mathrm {d}x -\lambda _{\varepsilon }\int _{B_{R/\varepsilon }}\phi _{\varepsilon }^{\perp }\frac{\partial A_{\varepsilon ,l}}{\partial x_m}\,\mathrm {d}x\nonumber \\&-\int _{B_{R/\varepsilon }}\frac{A_{\varepsilon ,l}^2}{H_{\varepsilon }^2}\frac{\partial A_{\varepsilon ,l}}{\partial x_m}(\psi _{\varepsilon }^\perp (q_l+x)-\psi _{\varepsilon }^\perp (q_l))\,\mathrm {d}x\nonumber \\&-\lambda _{\varepsilon }\int _{B_{R/\varepsilon }}\psi _{\varepsilon }^\perp (q_l)\frac{A_{\varepsilon ,l}^2}{H_{\varepsilon }^2}\frac{\partial A_{\varepsilon ,l}}{\partial x_m}\,\mathrm {d}x\nonumber \\&=o\left( \sigma \varepsilon ^2R_{\varepsilon }\sum _{j=1}^k\sum _{i=1}^2|a_{j,i}^{\varepsilon }|\right) . \end{aligned}$$

Then we study the algebraic equation for $a_{j,i}^{\varepsilon }$. Multiplying both sides of (10.12) by $\frac{\partial A_{\varepsilon ,l}}{\partial x_m}$ and integrating over $\mathbb {R}^2$, we obtain

$$\begin{aligned} r.h.s.&=\lambda _{\varepsilon }\sum _{j=1}^k\sum _{i=1}^2a_{j,i}^{\varepsilon } \int _{B_{R/\varepsilon }}\frac{\partial A_{\varepsilon ,j}}{\partial x_i}\frac{\partial A_{\varepsilon ,l}}{\partial x_m}\,\mathrm {d}x\nonumber \\&=\lambda _{\varepsilon }\sum _{j=1}^k \sum _{i=1}^2a_{j,i}^{\varepsilon }\delta _{jl}\delta _{im} \int _{\mathbb {R}^2}\left( \frac{\partial w}{\partial z_1}\right) ^2\mathrm {d}z\,(1+o(1))\nonumber \\&=\lambda _{\varepsilon }a_{l,m}^{\varepsilon }\int _{\mathbb {R}^2}\left( \frac{\partial w}{\partial z_1}\right) ^2\,\mathrm {d}z\,(1+o(1)). \end{aligned}$$

For the left hand side, we have

10.22 $$\begin{aligned} l.h.s.&=\sum _{j=1}^k\sum _{i=1}^2a_{j,i}^{\varepsilon }\int _{B_{R/\varepsilon }} \frac{A_{\varepsilon }^2}{H_{\varepsilon }^2}\left( \frac{\partial H_{\varepsilon }}{\partial x_i}-\psi _{\varepsilon ,j,i}\right) \frac{\partial A_{\varepsilon ,l}}{\partial x_m}\,\mathrm {d}x\nonumber \\&+\sum _{j=1}^k\sum _{i=1}^2a_{j,i}^{\varepsilon }\int _{B_{R/\varepsilon }}\varepsilon \mu '(|\varepsilon q_j|)\frac{x_i}{r}A_{\varepsilon ,j}\frac{\partial A_{\varepsilon ,l}}{\partial x_m}\,\mathrm {d}x+\int _{B_{R/\varepsilon }}\mathcal {I}_2\frac{\partial A_{\varepsilon ,l}}{\partial x_m}\,\mathrm {d}x. \end{aligned}$$

Using (10.17), we obtain

10.23 $$\begin{aligned} l.h.s.&=\sum _{j=1}^k\sum _{i=1}^2a_{j,i}^{\varepsilon }\xi \left( \frac{\partial ^2}{\partial {q_{l,m}}\partial q_{j,i}}F(\mathbf {q})\right) \, \int _{\mathbb {R}^2}w^2\frac{\partial w}{\partial z_m}z_m\,\mathrm {d}z\,\int _{\mathbb {R}^2}w^2\,\mathrm {d}z\nonumber \\&+\sum _{j=1}^k\sum _{i=1}^2a_{j,i}^{\varepsilon }\int _{B_{R/\varepsilon }}\varepsilon ^2 \mu ''(|\varepsilon q_j|)x_iA_{\varepsilon ,j}\frac{\partial A_{\varepsilon ,l}}{\partial x_m}\,\mathrm {d}x\nonumber \\&+o\left( \sigma \varepsilon ^2R_{\varepsilon }\sum _{j=1}^k\sum _{i=1}^2|a_{j,i}^{\varepsilon }|\right) . \end{aligned}$$

Note that in (10.23) there is no summation over *m*. From (10.22) and (10.23), we have

10.24 $$\begin{aligned} l.h.s.&=-c_1\xi \sum _{j=1}^k\sum _{i=1}^2a_{j,i}^{\varepsilon } \frac{\partial ^2}{\partial q_{l,m}\partial q_{j,i}}F(\mathbf {q})+c_2\varepsilon ^2\sum _{j=1}^k\sum _{i=1}^2a_{j,i}^{\varepsilon } \delta _{jl}\delta _{mi} \mu ''(|\varepsilon q_i|)+h.o.t., \end{aligned}$$

where $c_1,c_2$ have been introduced in (4.4) and (4.8), respectively. Combining the *l*.*h*.*s*. and *r*.*h*.*s*., we have

10.25 $$\begin{aligned}&\sum _{j=1}^k\sum _{i=1}^2a_{j,i}^{\varepsilon }\left( -c_1\xi \frac{\partial ^2}{\partial q_{l,m}\partial q_{j,i}}F(\mathbf {q})+c_2\varepsilon ^2\delta _{jl}\delta _{mi} \mu ''(|\varepsilon q_j|)\right) +h.o.t.\nonumber \\&\quad =\lambda _{\varepsilon }a^{\varepsilon }_{l,m}\int _{\mathbb {R}^2}\left( \frac{\partial w}{\partial z_1}\right) ^2\,\mathrm {d}z\,(1+o(1)). \end{aligned}$$

From (10.25), we see that the small eigenvalues with $\lambda _{\varepsilon }\rightarrow 0$ and are related to the eigenvalues of the $2k\times 2k$ matrix $M_{\mu }(\mathbf {q})$, where the $(j+ki,l+km)$-th component is the following

$$\begin{aligned} (M_{\mu }(\mathbf {q}))_{j+ki,l+km}= -c_1\xi \frac{\partial ^2}{\partial q_{l,m}\partial q_{j,i}}F(\mathbf {q})+c_2\varepsilon ^2\delta _{jl}\delta _{mi} \mu ''(|\varepsilon q_j|),\\ 1\le j,l\le k,1\le i,m\le 2. \end{aligned}$$

In Sect. 6 this matrix has been studied further and its eigenvectors and eigenvalues have been computed explicitly.
